# Quadra-Stable Dynamics of p53 and PTEN in the DNA Damage Response

**DOI:** 10.3390/cells12071085

**Published:** 2023-04-04

**Authors:** Shantanu Gupta, Pritam Kumar Panda, Daner A. Silveira, Rajeev Ahuja, Ronaldo F. Hashimoto

**Affiliations:** 1 Instituto de Matemática e Estatística, Departamento de Ciência da Computação, Universidade de São Paulo, Rua do Matão 1010, São Paulo 05508-090, SP, Brazil; 2 Condensed Matter Theory Group, Materials Theory Division, Department of Physics and Astronomy, Uppsala University, P.O. Box 516, SE-751 20 Uppsala, Sweden; 3 Children’s Cancer Institute, Porto Alegre 90620-110, RS, Brazil; 4 Department of Physics, Indian Institute of Technology Ropar, Rupnagar 140001, Punjab, India

**Keywords:** DNA damage response, PTEN, P53, cell fate determination, G2/M checkpoint, NSCLC

## Abstract

Cell fate determination is a complex process that is frequently described as cells traveling on rugged pathways, beginning with DNA damage response (DDR). Tumor protein p53 (p53) and phosphatase and tensin homolog (PTEN) are two critical players in this process. Although both of these proteins are known to be key cell fate regulators, the exact mechanism by which they collaborate in the DDR remains unknown. Thus, we propose a dynamic Boolean network. Our model incorporates experimental data obtained from NSCLC cells and is the first of its kind. Our network’s wild-type system shows that DDR activates the G2/M checkpoint, and this triggers a cascade of events, involving p53 and PTEN, that ultimately lead to the four potential phenotypes: cell cycle arrest, senescence, autophagy, and apoptosis (quadra-stable dynamics). The network predictions correspond with the gain-and-loss of function investigations in the additional two cell lines (HeLa and MCF-7). Our findings imply that p53 and PTEN act as molecular switches that activate or deactivate specific pathways to govern cell fate decisions. Thus, our network facilitates the direct investigation of quadruplicate cell fate decisions in DDR. Therefore, we concluded that concurrently controlling PTEN and p53 dynamics may be a viable strategy for enhancing clinical outcomes.

## 1. Introduction

The activation of one or both tumor suppressor pathways, such as p53/cyclin-dependent kinase inhibitor 1 (p21) and cyclin-dependent kinase inhibitor 2A (p16INK4A)/retinoblastoma protein (RB), is principally responsible for cellular senescence [1]. But such a mechanism is separate from quiescence, another condition of growth arrest (reversible cell-cycle arrest). Blagosklonny’s concept of “hyperfunction” [2,3] and “geroconversion” [4] is an excellent example of this. Conceptually, cellular senescence occurs in a two-phase process. At first, cells encounter a reversible cell-cycle arrest when the Mammalian target of rapamycin (mTOR) is inactive or “turned off”. Second, cells with stable activation or “on” of mTOR tend to a stable senescent growth arrest [2]. Thus, the choice between variable cell cycle arrest to stable senescence is determined by the mTOR pathway and this two-phase process is called geroconversion [3].

The cell cycle progression can be halted at any of the two checkpoints (G1/S and G2/M) by the tumor suppressor and cell cycle inhibitor p21, as is widely known [5]. Precisely, in the G2/M-phase, cells can be arrested by targeting either or both of the M-phase inducer phosphatase 3 (Cdc25c) and/or Cell division control protein 2 homolog (Cdc2) and G2/mitotic-specific cyclin-B1 (Cdc2-CyclinB) complex in DDR [5]. Within this frame, p21 induces cycle arrest by directly inhibiting the Cdc2-CyclinB complex [5]. Similarly, Wee1-like protein kinase (Wee1) is a crucial regulator of this checkpoint due to its ability to inhibit Cdc25C and the Cdc2-Cyclin B complex [6].

It is well-known that p53 controls PTEN in the DDR [7,8]. Despite this, recent research has revealed that Serine-protein kinase ATM (ATM) acts as an upstream controller of PTEN in the DDR, regulating cell fate choices such as cycle arrest and autophagy. Considering this, Park et al. [9] found that PTEN targets RAC-alpha serine/threonine-protein kinase (AKT) to generate p21 expression, which in turn causes halting cell cycle progression at the G2/M-phase in NSCLC. Likewise, Zhang et al. [10] revealed a positive connection between Wee1 and PTEN in Hepatocellular Carcinoma Cells (HCC). In more detail, Zhang and colleagues [10] found that PTEN mediates growth arrest at the G2/M-phase checkpoint by the activation of Wee1 by targeting AKT in DDR. While Chen and colleagues [11] uncovered a novel role of PTEN in autophagy induction in HeLa and A549 cell lines. In more detail, Chen et al. [11] uncovered that ATM phosphorylates PTEN on the serine site at 113, in addition to enabling PTEN nuclear translocation, which leads to autophagy through the phosphorylated-Transcription factor Jun (Jun)/Sestrin-2 (SESN2)/AMP-activated protein kinase (AMPK) (p-Jun-SESN2-AMPK) signaling pathways in A549 and HeLa cells. Collectively, these results show a complex signaling cascade induced by PTEN, triggering different phenotypic responses to its activation. As such, it demands new systems biology analysis to explore such a mechanism as well as the cooperation with p53 in the induction of cell fate control.

Non-coding RNAs (ncRNAs) with lengths higher than 200 nucleotides are referred to as long non-coding RNAs (LncRNAs). LncRNAs take part in a range of biological processes, including the advancement of the cell cycle, apoptosis, and genome stability [12]. Similarly, microRNAs (miRNAs) are tiny RNA molecules that are often connected to a range of biological processes, involving the development of tumors [13]. MiRNAs are well documented to serve as tumor suppressors or oncogenes in human cancer [14]. Taking this into consideration, Guo et al. [15] investigated the function of the lncRNA taurine-upregulated gene 1 (hereafter referred to as TUG1) and miR-221 in NSCLC progression in SPC-A1 and H520 cell lines, both SPC-A1 and H520 cell lines contain Wild-type (WT) p53. In more detail, Guo et al. [15] found that TUG1 was downregulated in NSCLC, while miR-221 was upregulated in corresponding cell lines. In addition, miR-221 directly targets PTEN expression. In this context, Guo et al. [15] found that TUG1 inhibits NSCLC proliferation through targeting of miR-221, it triggers growth arrest, senescence, autophagy, and apoptosis in SPC-A1 and H520 cell lines by stimulating PTEN at the G2/M phase. It is usually accepted that p53 induces PTEN in DDR in cancer cells [7,8]. Interestingly, TUG1 is also regulated by p53 in NSCLC [16]. However, it is currently unclear how these two p53-inducible proteins, TUG1 and PTEN, regulate cell fate choices at the G2/M phase.

The primary purpose of modeling a complicated system, like the quadra-stable gene network of p53 and PTEN signaling in DDR, is to develop a model that can quantitatively predict each component’s outcome. As a result, Boolean network modeling is the best method for combining existing information into a logical framework that is compatible with experimental results [17]. Signaling components (signaling proteins, and ncRNAs such as lncRNAs, and miRNAs) are described as nodes, and interconnections between them are defined as edges [18,19,20,21,22]. Cell fates are coupled with model attractors (fixed points or cyclic attractors) whose designation and reachability qualities are particularly suited to this approach [23]. Thus, dynamic Boolean network modeling is the finest option to explore the dynamic behavior of the system [24,25,26,27]. More details about Boolean modeling can be found in the “Materials and Methods” section.

As a result, we intend to propose a dynamic Boolean model for G2/M checkpoint regulation based on the data provided above. The goal of this research was to investigate cell fate decisions mediated by p53 and PTEN by considering four phenotypes: cell cycle arrest, senescence, apoptosis, and autophagy. According to our knowledge, this is the first dynamic Boolean network model applied in the investigation to assess the four phenotypes in DDR at the G2/M checkpoint. The finding of switch-like behaviors of the molecular states in such quadra-stable dynamics might provide new insights into the cell fate determination triggered by p53 and PTEN. The investigation of such behaviors may assist us in comprehending the biological processes that lead to cell fate decisions.

## 2. Materials and Methods

### 2.1. Simulations of the Boolean Network Model and Encoding of PubMed Articles into Boolean Rules

The Boolean methodology is founded on the regulatory graph definition, where a signaling component is represented by a node, and the relationship between two nodes is described by edges (by activation or inhibition).

Boolean network models are full of dynamic presentations of a signaling system in which 2 possible states describe each node: on or 1 (interpreted as active), and off or 0 (interpreted as inactive). For instance, the ON state (activation) of the PTEN protein is associated with phosphorylation, this means that there is an acceptable amount of phosphorylation by p53 or ATM, which can trigger PTEN expression in the DDR. In our dynamic model, we constituted 29 signaling components including proteins, lncRNA TUG1, and miR-221 because they are known to regulate DNA damage repair signaling pathways based on information in the literature. Accordingly, in our model, there is one input named “DNA damage”. In Table S1, We provide an extensive list of Boolean functions for nodes, as well as their regulators and references. A complete list and description of the Boolean functions employed are in Table S2.

In this modeling approach, the simulation of succeeding occurrences is implemented by update schemes. We employed a non-deterministic (stochastic) update strategy in which individual node is updated randomly [28]. Attractors are the final outcome of Boolean model simulations. A state transition graph (STG) may decode the Boolean network models’ dynamic execution. The STG executes all possible routes from a single beginning state to a final state. A collection of evolutions trapped within a constrained set of states in the STG denotes a cyclic state, whereas terminal nodes without outgoing edges are referred to as fixed points (or stable states or endpoints). To further study the influence of individual nodes on network dynamics and the consequent phenotype, we test node values to be 0 or 1, respectively, employing Gain-of-Function (GoF) or Loss-of-function (LoF) in-silico perturbations [28].

### 2.2. Gene Regulatory Network Evolution, as Well as Public Database and Tool Sets

**Figure 1:**
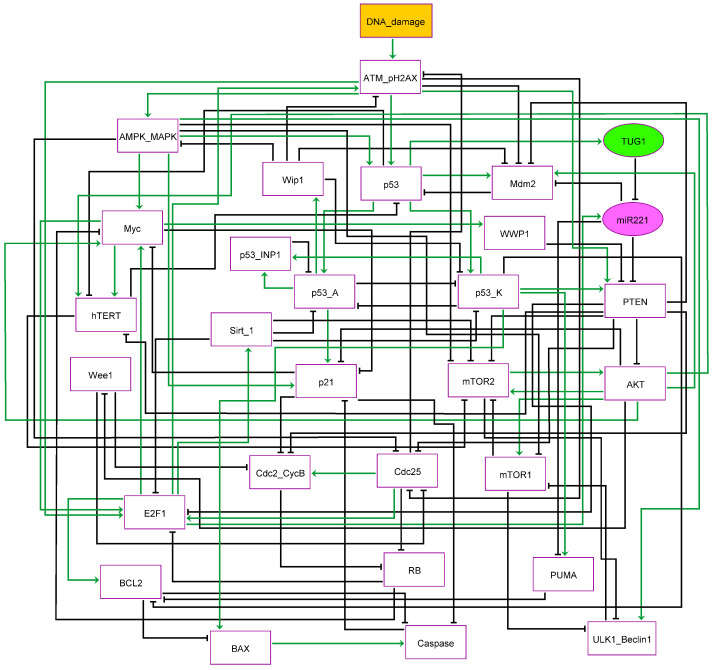
**The network of quadra stable dynamics of p53 and PTEN caused by DNA damage.** Green edges with arrowheads demonstrate positive interactions, whereas black edges with hammerheads indicate negative interactions, respectively. Node colors define their function as follows: DNA damage as input in the orange node, signaling proteins are rectangular (white nodes), lncRNA TUG1 in an oval node (green), and miR-221 in an oval node (pink). Table S1 contains the logical rules governing the nodes and their activity, while Table S2 gives the full names of the network elements associated with each node and the biological justifications for the edges and their regulators.

To build a quadra stable gene regulatory Boolean model of p53 and PTEN in DDR, we only used databases like PubMed Probe and BioGRID 3.5 (https://thebiogrid.org/) [29]. Emphasis was placed on selecting genes or proteins including the lncRNA TUG1 and miR-221 that are involved in the DDR signaling pathway (Figure 1). Furthermore, such as proteins that are directly targeted through miR-221 have been identified by using bibliographic databases like; miRTargetLink [30] and TargetScan [31].

The framework of the Boolean network model and the simulation of the advancements were handled using the java-based program GINsim 3.0.0b, which is voluntarily unconstrained to academics and accessible at (http://ginsim.org/downloads) [32]. The algorithms of GINsim demonstrate all the terminal nodes (attractors) for the wild-type systems and also for diverse mutant cases [32]. Additionally, GINsim helps measure the chances of obtaining particular attractors. We employed the Monte Carlo technique with exact exit probabilities [28] in this investigation. In the “Code availability” section, you may find the model file.

## 3. Results

### 3.1. The G2/M Checkpoint’s Proposed Molecular Mechanism

Stress-induced activation of the G1/S or G2/M-phases in the DDR has been thoroughly studied [33]. Cycle arrest or senescence for repair, autophagy, or apoptosis can be induced at both checkpoints [34,35,36]. ATM is activated by DNA double-strand breaks (DSB) [37]. ATM phosphorylation regulates the activation of downstream p53. In the NSCLC cell line, the TUG1/miR-221 axis is critical for the induction of the G2/M-phase, which we emphasize here. NSCLC cells with the knockout of TUG1 or downregulated or overexpression of miR-221 could not control cell cycle progression to the G2/M phase. We illustrate our Boolean network model in interactions implicated in the induction of G2/M-phase by DNA damage, including the TUG1/miR-221 axis [15]. There are 29 nodes in the model that are represented by proteins, lncRNA, and miRNA, and 91 direct connections between them.

There is one input in our model called “DNA damage” (Figure 1). Upon DNA damage, ATM initiates p53 expression. Activated p53 causes the activation of the E3 ubiquitin-protein ligase Mdm2 (MDM2), consequently, it directs the down-regulation of p53 [38]. Our model is based on the multiple phosphorylation modes of p53 and is distinguished by additional nodes: p53 is associated with Mdm2 through a positive interaction that is needed to initiate p53-Arrest (p53-A) and p53-Killer (p53-K) [39]. p53-A pinpoints p53, phosphorylated at Ser-15 and Ser-20, whereas p53-K shows that p53 is more phosphorylated at Ser-46 which regulates autophagy or apoptotic cell death. The modification between p53-A and p53-K is controlled by Protein phosphatase 1D (Wip1) and Tumor protein p53-inducible nuclear protein 1 (p53-INP1) [40]. p21, Wip1 and tumor protein p53 p53-INP1 form p53-A [40,41]. The Cdc2/Cyclin B complex is directly suppressed by p21 [42]. In this way, p21 segregates Cdk1 into the cytoplasm and directly inhibits the actions of Cdc25c [43], leading to G2/M arrest [42]. Besides, AKT is a negative regulator of p21 activity [44], PTEN induces G2/M cycle arrest by directly inhibiting AKT [9], resulting in the activation of p21. Likewise, PTEN directly targets AKT which triggers the activation of Wee1 and results in growth arrest at the G2/M-phase [10]. Whereas p53-K directly inhibits Apoptosis regulator Bcl-2 (Bcl2). While, p53-K positively interacts with Bcl-2-binding component 3 (PUMA), Apoptosis regulator BAX (BAX), and caspase-3, which triggers apoptosis in NSCLC. p53-K activates PTEN expression to down-regulate AKT/mTOR signaling pathway.

Additionally, the G2/M-phase checkpoint activation results in the activation of phosphorylated histone H2AX (γ−H2AX) and the ATM pathway. This is followed by the induction of Serine/threonine-protein kinase Chk2 (Chk2) which inactivates CDC25C, resulting in G2/M arrest. In this context, Zhang et al. [45] found that PTEN regulates the γ−H2AX activity and enhanced DNA damage into the cells by phosphorylating Cdc25C, which triggers the activation of the ATM/Chk2 axis and thus arrest cell cycle progression at the G2/M-phase checkpoint. Therefore, in the network, ATM-H2AX is shown in a single node. Similarly, AMP-activated protein kinase (AMPK) and Mitogen-activated protein kinase (MAPK) are in the same node, both of which can be induced by ATM in the DDR [46,47]. While Wip1 is a negative regulator of MAPK activity [48]. Both AMPK and MAPK can induce p53 expression [49,50]. Additionally, both MAPK and AMPK contribute to G2/M-phase cell cycle arresting by directly inhibiting Cdc25 expression [50,51]. Furthermore, MAPK and AMPK can directly trigger the activation of Serine/threonine-protein kinase ULK1 (ULK1) expression [52,53], while both are the negative regulator of mTOR activity [53,54].

Furthermore, ATM also regulates PTEN expression [11]. In more detail, it was recently found a direct positive interaction between ATM and PTEN and reported that ATM induced autophagy by activation of PTEN and AMPK pathways [11], which directly inhibits mTOR 1/2 leading to the activation of the ULK1/Beclin 1 complex (in our model, the ULK1/Beclin1 complex is shown as a single node [55]). Thus, PTEN-inducible autophagy is ATM/AMPK pathway-dependent [11].

We presented our Boolean network model of G2/M-phase modulation in NSCLC based on the above important connections (Figure 1).

### 3.2. Simulations Indicate Quadra-Stable Dynamics of the Model

**Figure 2:**
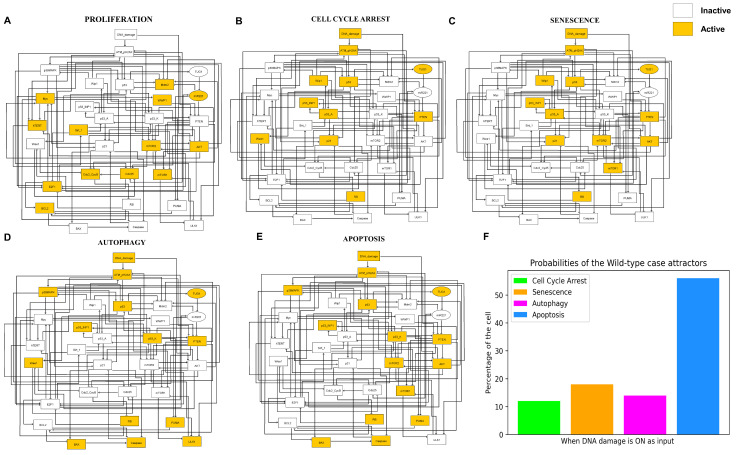
**Dynamics of Wild-Type Case of the quadra-stable gene network model.** Each figure of the model illustrates a stable attractor. Orange and white color nodes specify the state of the respective molecule i.e., ON/Activation and OFF/Inactivation, respectively. We found 5 fixed points also known as (stable states). Each fixed point describes a phenotype according to the activation of the signaling component involved. In one out of these five fixed points, we found the lack of DNA damage, which serves as the network’s input. While the other four fixed points are in the existence of DNA damage also known as quadra-stable dynamics or quadra-stability (when the input of the model is “ON”). Here we explain all these fixed points. (**A**) The stimulation of cell cycle markers in the absence of DNA damage (Input) reflects a Proliferation phenotype. The other four fixed points are in the existence of DNA damage. (**B**) The activation of p53, p53-A, p21, and Wip1 in DDR represents the Cell cycle arrest phenotype. (**C**) While the activation of mTORC1/2 along with p53-A, and p21 defines the senescence phenotype. (**D**) The activation of ULK1/Beclin 1 complex jointly with p53-K, Puma, Bax, and Caspase describes the autophagy phenotype. (**E**) Whereas, the activation of p53-K, Puma, Bax, and Caspase indicate apoptosis phenotype. (**F**) The possibilities of each attractor or phenotype of the wild case in DDR were obtained by Monte-Carlo simulation (100,000 Runs).

In Figure 2, we denote the wild-type case of the network. We obtained 5 fixed points (also called steady state). The orange and white colored nodes mean activation and deactivation of the respective molecules, respectively. In this way, we can specify which molecules are involved in each fixed point. There is only one input to our network which is called DNA damage. This input to the network can be “ON/Active” or “OFF/Inactive”. Of the five fixed points, one fixed point, we found, was when the input was turned off. The remaining four fixed points are also called quadra-stable dynamics, which we found in the existence of DNA damage (when the input is “ON”). In more detail, Figure 2A shows a proliferative steady state in the lack of input (when the DNA damage is “OFF”). As can be seen in Figure 2A, only cell cycle regulators are occupied, whereas tumor suppressors and cell cycle inhibitors are non-functioning. The remaining fixed points are in the existence of DNA damage. In more detail, Figure 2B, activation of p53-A with p21 defines cell cycle arrest. While Figure 2C, p53-A shows senescence phenotype due to activation of mTOR1/2 with p21. Figure 2D,E mark fixed points of cell death: autophagy and apoptosis, respectively. In summary, Figure 2D represents the autophagy phenotype due to the induction of ULK1 as well as p53-K, BAX, and Caspase-3. While Figure 2E describes the apoptotic phenotype without ULK1 due to the activation of p53-K, BAX, and Caspase-3. It is a non-deterministic (stochastic) manner of quadra-stability network dynamics where four distinct fixed points (when the input to the network is “ON”) are determined at random from the precise initial position. The odds of these fixed points are not always comparable. Using Monte Carlo simulations (100,000 runs), we identified, 12% for cycle arrest, 18% for senescence, 14% for autophagy, and 56% for apoptosis in DDR, as shown in Figure 2F.

Interestingly, this quadra stability that we reported above is in excellent agreement with Guo et al. [15] Which we describe in the next section.

### 3.3. A Network-Guided Quadra Stability Correlation with Experimental Outcomes in DDR

Recently, Guo et al. [15] investigated the function of TUG1 in NSCLC. These authors additionally showed that upregulation of TUG1 targets miR-221 and induces PTEN expression, which inhibits proliferation by induction of growth arrest, senescence, apoptosis, and autophagy in NSCLC [15]. To test whether our network can produce similar results according to Guo et al. [15], we carried out single and double node perturbations corresponding to those demonstrated by Guo et al. [15].

**Figure 3:**
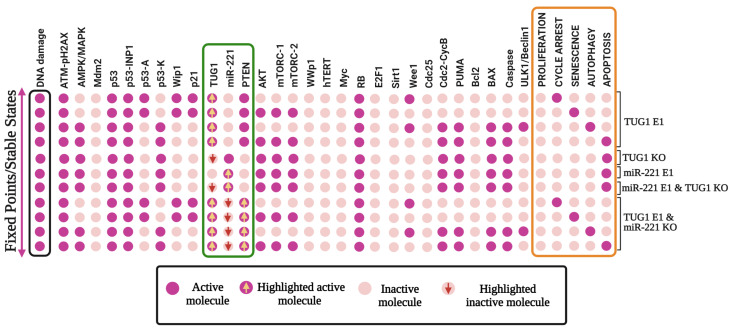
**Model Validation-1.** The GoF and LoF perturbations are consistent with those used in Guo et al. [15]. Gain of function (GoF) is shown by ectopic expression (E1), whilst loss of function (LoF) is indicated by knockout (KO) of the same network component. Light pink cells mark inactivation (OFF), while pink cells mark activation (ON). Steady states are determined for specific modeling scenarios: TUG1 E1, TUG1 KO, miR-221 E1, miR-221 E1 with TUG1 KO, and TUG1 E1 with miR-221 KO. The input DNA damage is marked in a black box in the left column, and the model outputs are highlighted in an orange box in the right column: proliferation, cell cycle arrest, senescence, autophagy, and apoptosis. Each line signifies a single fixed point or steady state associated with the input.

The results are shown in Figure 3. Overexpression (E1) of TUG1 triggers growth arrest, senescence, autophagy, and apoptosis. Whereas its knockdown abolishes growth arrest, senescence, and autophagy, but only causes apoptotic cell death. Next, overexpression of miR-221 abrogates growth arrest, senescence, and autophagy, but only induces apoptotic cell death. Subsequently, overexpression (E1) of miR-221 jointly with knockdown (KO) of TUG1 causes apoptotic cell death. In conclusion, Guo et al. [15] reported that knockdown (KO) of miR-221 together with overexpression (E1) of TUG1 directs to increased PTEN expression, which triggers growth arrest, senescence, autophagy, and apoptosis at the G2/M-phase. As we can see in Figure 3, our outcomes are in exceptional agreement with the results of Guo et al. [15].

### 3.4. Comparison of Experimental Observations with Phenotype Probabilities

Beside, for individual node perturbations, we chose the phenotypic probability, parallel to what was observed by Guo et al. [15]. This was directed by perturbing the GoF-LoF of each individual molecule, followed by 100,000 Monte Carlo simulations performed with the input employed. Using the gain of function of TUG1, as performed by Guo et al. [15]. They examined the role of this TUG1 in response to cisplatin (DDP), in which it induced growth arrest, senescence, apoptosis, and autophagy of SPC-A1 and H520 cells.

**Figure 4:**
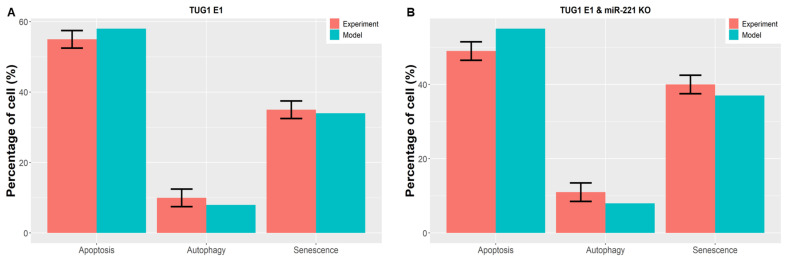
**Model Validation-2.** Investigation of model-predicted probabilities in DDR for Autophagy, Apoptosis, and/or Senescence with Guo et al. [15]. The red bar depicts experimental data for SPC-A1 and H520 cells, while the green bar corresponds to model probabilities. (**A**) In DDR, ectopic expression of TUG1 (E1) induces apoptosis (58%), autophagy (8%), and senescence (34%). (**B**) Overexpression (E1) of TUG1 with knockdown (KO) of miR-221 induces apoptosis (55%), autophagy (8%), and senescence (37%). As experimental observations [15], the mean ± standard deviation of three biological replicates is presented.

Worth noting is that Guo et al. [15] provided quantitative data for cell cycle arrest but not senescence. Therefore, we combined the cell cycle arrest + senescence data achieved by our network and compared it with the cell cycle arrest obtained from Guo et al. [15], which we illustrated as a senescent phenotype. The results are shown in Figure 4. Overexpression (E1) of TUG1 triggers autophagy, apoptosis, and senescence. We found that forced overexpression (E1) of TUG1 could produce 55% apoptosis, 10% autophagy and 34% senescence (11% cell cycle arrest + 23% senescence). See Figure 4A. Subsequently, overexpression of (E1) TUG1 with knockdown (KO) of miR-221 (see Figure 4B) deliver 49% apoptosis, 11% autophagy and senescence 37% (cell cycle arrest 10% + senescence 27%). As illustrated by Figure 4A,B, our system has a good agreement with Guo et al. [15].

### 3.5. Quadra Stable Dynamics Is Regulated by p53 in DDR

**Figure 5:**
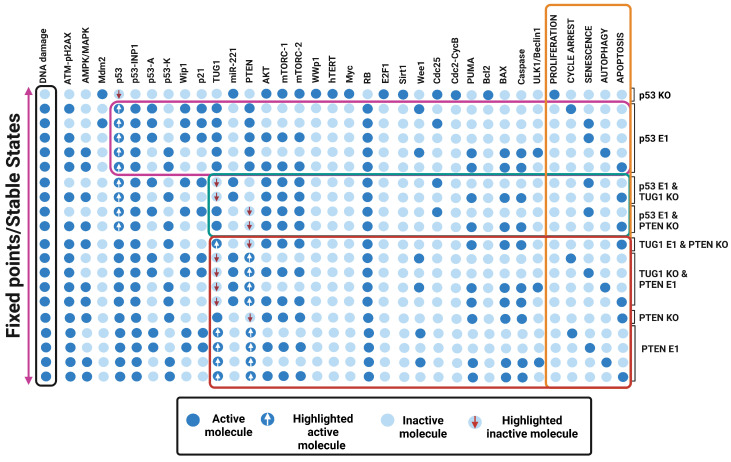
**Molecule perturbation by gain-and-loss-of-function (GOF/LOF).** Ectopic/overexpression expression (E1) illustrates the gain of function and loss of function of knockout (KO) of the same network element. Light blue cells signify inactivation (OFF), while blue cells characterize activation (ON). Steady states are chosen for specific modeling scenarios: p53 KO, p53 E1, p53 E1 together with TUG1 KO, p53 E1 along with PTEN KO, TUG1 E1 with PTEN KO, TUG1 KO with PTEN E1, PTEN KO and PTEN E1. The input DNA damage is shown in the queue on the left, emphasized in a black box and the right-most queue demonstrates the model outcomes, highlighted in an orange box: proliferation, cell cycle arrest, senescence, autophagy, and apoptosis. Each line denotes a specific input-related fixed point or steady state.

It is generally known that the tumor suppressor p53 coordinates cell fates more efficiently in DDR. TUG1 and PTEN have both been found to be important downstream targets of p53 in NSCLC.We, therefore, set out to investigate whether p53 contributes to quadra-stability in DDR. For that, we carry out perturbations of p53. As can be seen in Figure 5, highlighted in the pink box, Overexpression (E1) of p53 induces quadra stability in DDR. i.e., cell cycle arrest, senescence, autophagy, and apoptosis. Whereas, its knockdown abrogated all these phenotypes and caused proliferation. Our findings are in order with those of Park et al. [56], who have recently established a critical function of p53 in NSCLC cells in DDR.

### 3.6. Perturbations of TUG 1 and/or PTEN Can Break p53-Induced Quadratic Dynamics in DDR

The abovementioned findings imply that p53 controls quadra-stable dynamics in DDR. Nevertheless, as claimed by Guo et al. [15], this quadra stability is controlled by the TUG1/PTEN axis. Therefore, it remains to be clarified whether TUG1 or PTEN is required to regulate quadra stability in the DNA damage response. Consequently, we chose to knockdown (KO) perturbation TUG1 and/or PTEN with overexpression (E1) of p53. In this manner, the biological roles of these two downstream targets of p53 in NSCLC, TUG1, and PTEN, may be established. The results are shown in Figure 5, highlighted in the green box. First, we demonstrate overexpression (E1) of p53 together with knockdown (KO) TUG1. Surprisingly, cell cycle arrest and autophagy are abrogated, but senescence and apoptosis are not. Likewise, overexpressing (E1) p53 and knocking down (KO) PTEN result in the cancellation of autophagy and cell cycle arrest. These results indicate that both or one of them (TUG1 and/or PTEN) may control cell cycle arrest and autophagy, but senescence and apoptosis are controlled by p53 at the G2/M-phase checkpoint in NSCLC.

### 3.7. “A New Guardian of Genome-PTEN” Is Required to Maintain the Quadra-Stable Dynamics of p53 in DDR

To explore further molecule mechanisms involving cell cycle arrest and autophagy by the TUG1 and/or PTEN, We conducted knockdown (KO) and overexpression (E1) perturbations among them. In more detail, first, we performed perturbation overexpression of TUG1 and knockdown of PTEN. As can be seen in Figure 5, highlighted in the red box, it induces only apoptosis phenotype. Next perturbation, overexpression (E1) of PTEN, and knockdown (KO) of TUG1 inhibit proliferation by the induction of quadra stability i.e., cell cycle arrest, senescence, autophagy, and apoptosis. These findings clearly demonstrate that PTEN is necessary to sustain p53’s quadra stability dynamics. Our results have a significant implication: PTEN is a molecular switch that can govern cell cycle arrest and autophagy, whereas p53 is the molecular switch that regulates senescence or apoptosis.

### 3.8. Consistency among Model Attractors and Other Experimental Investigations

To combine the network for NSCLC cells with additional cell types, we accomplished a comprehensive bibliographic search regarding the effect of p53 and PTEN on the cell destiny of different cell types. We evaluated tumor cells expressing p53 in its wild-type with downregulated PTEN expression at the G2/M-phase checkpoint. Furthermore, we decided to remove TUG1 and miR-221 in this analysis because of their abnormal expression in various cancers. In this way, we can outline the dynamic roles of tumor suppressors of p53 and PTEN in cell fate decisions.

In such a manner, we located that experimental studies such as for HeLa cells [11,57] and MCF-7 cells [45,58]. The leading direction of all these investigations is to demonstrate that activated PTEN inhibits tumor development by improving the p53 pathway and selection of cell fate at the G2/M-phase [11,45,57,58]. Table 1 shows the understanding of our in-silico outcomes and the in-vivo and in-vitro results. Indeed, we emphasized the individual role of p53 and/or PTEN in cell fate decisions. For that, first, we show the perturbation of overexpression (E1) of p53 combined with the knockdown (KO) of PTEN. Then, in similar cell lines, we show only overexpression (E1) of PTEN. In this way, we can underline the specific role of p53 or PTEN in phenotypes. For instance, in breast cancer cell lines (MCF-7), we executed the perturbation among p53 and PTEN i.e., overexpression (E1) of p53 and the knockdown (KO) of PTEN, we found that p53 triggers senescence and apoptosis at the G2/M-phase checkpoint, which is in agreement with Huang et al. [59]. Whereas PTEN is activated in similar cell lines i.e., overexpression (E1) of PTEN induces cell cycle arrest and autophagy at the G2/M-phase checkpoint. which is in accordance with Zhang et al. [45], Arafa et al. [58] and Rovito et al. [60]. As can be seen in Table 1, our network represents a fine agreement with these studies [11,45,57,58]. Therefore, we suggest that p53 and PTEN may similarly affect cell fate decisions in HeLa and MCF-7 cell lines. Once the model results match the experimental studies, We can suggest some predictions about single or double-node perturbation, which were not located in the literature (see question marks in Table 1).

**Figure 6:**
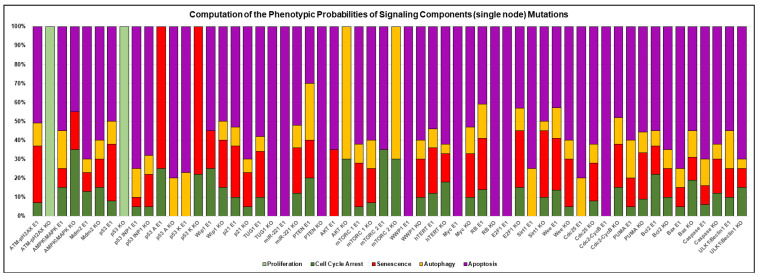
**Probability test of the Boolean network model’s signaling component (single node) mutation.** The GoF is represented by ectopic/overexpression expression (E1), while the LoF is defined by knockout (KO), respectively.

Additionally, we conducted a rigorous analysis of the possibilities revealed by disrupting individual nodes to pinpoint single-node effects on the quadra-stable dynamics of p53 and PTEN (see Figure 6). Using this strategy, we see that some perturbations may precisely differ in the occurrence of a particular phenotype, senescence was accelerated by Myc or Sirt-1 knockdown. However, Rb knockdown or overexpression of WWP1 or E2F1 amplified apoptosis. Similar findings are obtained by inhibiting AKT or mTORC2, which enhances cell cycle arrest and autophagy. Our results imply that these signaling components can regulate the possible consequences of cell fate.

## 4. Discussion

The above-defined findings have a few key characteristics which are important to highlight. In the following, we provide a brief discussion thereof.

### 4.1. Influence of p53 on TUG1/miR-221 Axis and Its Consequences on PTEN Impulsivity in DDR

In this present investigation, we analyzed molecular mechanisms related to TUG1 and miR-221, and PTEN at the G2/M-phase in NSCLC (see Figure 1). Recently Guo et al. [15] have described a unique method of cycle arrest, senescence, autophagy, and apoptosis by the TUG1 influence on PTEN function by targeting miR-221 in NSCLC. In more detail, Guo et al. [15] found that TUG1 was downregulated in NSCLC, whereas miR-221 was upregulated in the respective cell lines. Furthermore, miR-221 directly targets PTEN expression. In this context, Guo et al. [15] uncovered that TUG1 inhibits NSCLC proliferation by targeting miR-221, which triggers activation of PTEN and induced cell cycle arrest, senescence, autophagy, and apoptosis at the G2/M-phase checkpoint. We have tested whether our model can produce similar results according to Guo et al. As we can see in Figure 3, our observations are in perfect agreement with those obtained by Guo et al. [15].

Additionally, we compared single/double node perturbation probabilities of phenotype, which was accomplished by Guo et al. [15]. However, Guo et al. [15] did not provide quantitative data for senescence. It might be possible that they only obtained cell cycle arrest in the early stage, which caused senescence later, as suggested by Blagosklonny’s theory of “geroconversion” [4]. Accordingly, we integrated the cell cycle arrest + senescence data gained by our network and compared it with the cell cycle arrest conveyed by Guo et al. [15]. Thus, we found a quantitative comparison between the autophagy, apoptosis, and senescence phenotypes. The results can be seen in Figure 4. We found that TUG1 (E1) overexpression triggers autophagy, apoptosis, and senescence, see Figure 4A. Next, we used double-node perturbation (Figure 4B), that is, we overexpressed (E1) TUG1 with knockdown (KO) of miR-221. As can be seen in Figure 4, our model provides a very good arrangement.

It is well known that in NSCLC both TUG1 and PTEN are controlled by p53 [7,8,16]. Likewise, ATM also regulates the activity of PTEN in NSCLC [11]. However, Guo et al. [15] neglected the dynamic role of p53 in NSCLC. Therefore, we produced a dynamic Boolean framework that links the p53 and PTEN pathways to quadra-stable dynamics, including cell cycle arrest, senescence, autophagy, and apoptosis in cancer cells. In terms of biology, our model promotes cell cycle arrest because p21 and p53-A are stimulated, and it generates senescence because p21, p53-A, and the mTOR pathway are enabled (when serine 46 on p53 is not phosphorylated) [39]. Whereas p53-K defines the activation of apoptosis and autophagy (when serines 15, 20, as well as 46 of p53, become phosphorylated) [39].

The dynamics of p53 in DDR have been extensively studied; see a recent review by Wang et al. [62]. Previously, inspired by Zhang et al. [39], we have demonstrated that the p53 system is connected in the switchable dynamics between cycle arrest and apoptosis in cancer cells [18,19,21,34,35,36]. Lately, we added a new phase of p53 dynamics, i.e., the tristable dynamics of p53 in DDR [20,63]. More specifically, in U-87 and HeLa cell lines, we showed the critical involvement of p53 in senescence, apoptosis, and the activation of autophagy (via microRNA-16 (miR-16), microRNA-34a (miR-34a), and microRNA-449a (miR-449a)) [20,63].

Following that, we established the first dynamic Boolean network of DNA damage-mediated G2/M-phase in NSCLC to evaluate cell fate decisions amongst the four phenotypes of cell cycle arrest, senescence, autophagy, and apoptosis.

### 4.2. PTEN and Quadra-Stable Dynamics of p53 in DDR

**Figure 7:**
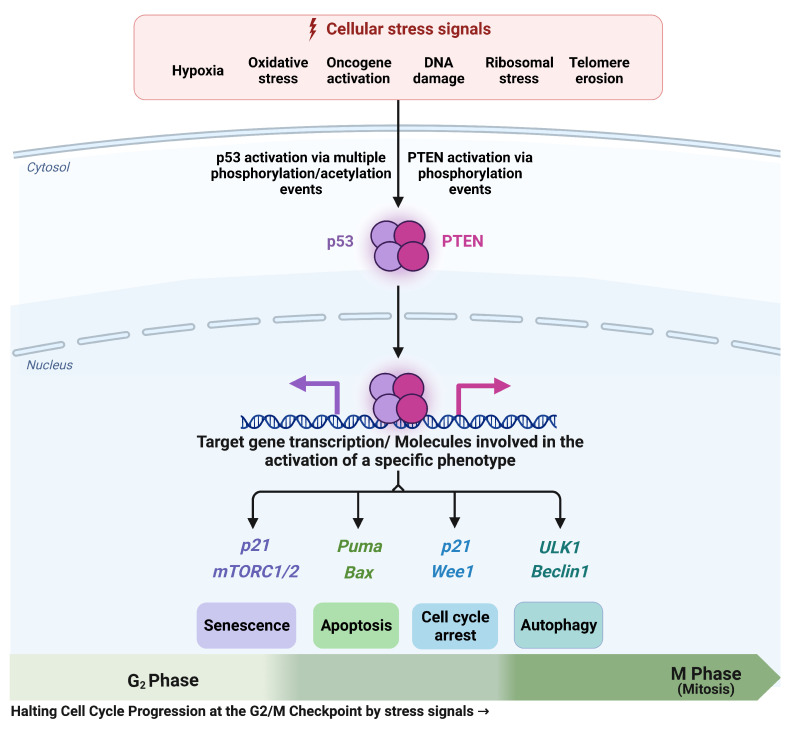
**Quadra-stable gene network of p53 and PTEN in response to DNA damage or stress signals.** P53 and PTEN can be activated individually in response to stress signals or DNA damage through phosphorylation/acetylation. Additionally, p53 can directly activate PTEN transcription. Once p53 or PTEN are activated, they can trigger various cellular outcomes at the G2/M-phase checkpoint. Senescence is induced by p53 via the p21 and mTOR pathways. p53, on the other hand, triggers apoptosis by upregulating BAX, PUMA, and Caspase-3. While, PTEN inhibits AKT, which causes cycle arrest by increasing p21 and Wee1, and facilitates autophagy by suppressing the AKT/mTOR pathway, which triggers the installation of ULK1/Beclin-1. Consequently, the four possible phenotypes of cell cycle arrest, senescence, autophagy, and apoptosis (quadra-stable dynamics) in the DDR are ultimately regulated by p53 and PTEN.

It is commonly known that p53 is referred to as the “guardian of the genome” due to its crucial role in safeguarding DNA integrity [64]. Similar to p53, PTEN has also been determined as the “new defender of the genome” [65] for its potential to prevent the proliferation and survival of cells by initiating DNA repair mechanisms, which involve targeting the p13K/AKT pathway [65]. In the current study, we were able to determine the switchable molecular mechanisms regulated by PTEN and p53. As can be seen in Figure 5. Overexpression of p53 regulates quadra-stability and PTEN is required to maintain this quadra-stability in DDR. Without PTEN, p53 regulates only bistability, i.e., senescence and apoptosis as previously shown by Zhang et al. [39]. At the G2/M phase, we uncovered that PTEN is required to trigger cell cycle arrest and autophagy. In summary, p53 regulates bistable dynamics between senescence and apoptosis, whereas additional bistable dynamics between cell cycle arrest and autophagy are controlled by PTEN. When analyzed collectively, p53 and PTEN represent quadra-stable dynamics in the DDR, see Figure 7. All of these remarkable findings, however, are dependent on a discrete basis of model components. One of our method’s shortcomings is its inability to predict time-dependent attributes and the precise evolution of expression levels over time.

In the coming sections, we will briefly discuss how these two tumor suppressors and the “protectors of the genome” affect cell fate determinants in the DDR during the G2/M phase.

#### 4.2.1. Senescence and Apoptosis Induced by p53

In cancer cells, p53 is a key regulator of senescence and apoptotic cell death [18]. In NSCLC, recently Chen et al. [66] revealed that p53 directly inhibits Telomerase reverse transcriptase (hTERT) expression, which triggers senescence in DDR in NSCLC. In more detail, Chen and colleagues revealed that p53 suppressed hTERT function in the H460 and H1299 cell lines. Indeed, Chen et al. [66] emphasized the essential role of p53 by targeting hTERT in senescence activation. Similarly, Guo et al. [15] showed that PTEN inhibits hTERT activation, which initiates the senescent process. However, in our work, we found that senescence was regulated by p53. Additionally, according to Blagosklonny’s theory of “hyperfunction” and “geroconversion”, senescence is a two-step process that requires the stimulus of the mTOR pathway or in other words, the mTOR pathway is a switch between the cell cycle arrest and senescence, when the mTOR pathway is turned OFF and p21 is turned ON, cells proceed to cell cycle arrest. Whereas, when p21 is activated as well as the mTOR pathway is activated, cells experience senescence. Interestingly, through our network, we were able to capture two-step senescence. which has recently been observed by Kim et al. [67] in EJ and H1299 cell lines.

The p53-induced apoptosis is based on the inhibition of BCL2, which triggers the activation of PUMA, BAX, and Caspase activation in NSCLC [68].

#### 4.2.2. Cell Cycle Arrest and Autophagy Is Regulated by PTEN

Suppression of Cdc25C is coupled to the cell cycle arrest at the G2/M-phase owing to its capacity to stimulate and modulate the Cdc2/CycB complex [69], which triggers cell cycle arrest in DDR [69]. In this manner, recently, it was revealed that PTEN effectively suppresses Cdc25C [45] as well as the Cdc2/CycB complex [70]. While, indirectly, through the activation of p21 and/or Wee1. In more detail, Wee1 is a key controller of the G2/M-phase, Wee1 selectively inhibits the Cdc25C and Cdc2/CycB complex in DDR [10]. PTEN directly targets AKT, which initiates Wee1 and causes the arrest of the cells at the G2/M phase. Similarly, PTEN is also enhanced p21 expression by the inactivation of ATK [9]. In more detail, p21 is a well-known cell cycle suppressor and tumor suppressor. It is well determined that p21 is coordinated by p53 and has the ability to impede cell cycle progression at both the G1/S and G2/M phases [5]. Interestingly, p21 expression is negatively controlled by AKT and PTEN directly inhibits AKT, which causes upregulation of p21 expression [9]. In this way, PTEN controls cycle arrest during the G2/M phase [9,10,45,70]. For more detail about the cell cycle regulation by PTEN (see the review by Brandmaier [71]).

PTEN is associated with autophagy induction in DDR through mTOR1/2 and the ULK1/Beclin 1 complex. More precisely, new research [11] demonstrates that ATM regulates the expression of PTEN at the serine 113 level, facilitating PTEN nuclear export. PTEN nuclear export drives autophagy in A549 and HeLa cells through the AMPK pathway. ATM-mediated autophagy is thus triggered via the PTEN/AMPK pathway [11].

### 4.3. Implications of Quadra-Stable Dynamics of p53 and PTEN in Cancer Treatment

The finding of quadra-stable dynamics of p53 and PTEN in the DDR of NSCLC provides a new way of understanding the mechanisms of DDR. We found that PTEN and p53 have distinct roles in DDR. PTEN induces cell cycle arrest and autophagy, while p53 triggers senescence and apoptosis. As a result, DDR leads to a quadra-stable dynamic that is governed by these two proteins. This finding has important implications for cancer therapy development. By simultaneously regulating the dynamics of PTEN and p53, the effectiveness of treatment can be improved. Furthermore, these findings can be used to guide the development of new cancer therapies that target the TUG1 and miR-221 axis. In conclusion, our findings demonstrate the importance of PTEN and p53 in DDR and suggest that regulating their dynamics may lead to improved therapeutic outcomes. This research provides valuable insight into DDR and has the potential to revolutionize the way cancer is treated in the future.

## Figures and Tables

**Table 1:** Agreement between our Boolean network model and in-vivo/in-vitro investigations in different cell lines. Both ectopic/overexpression (E1) and knockout (KO) characterize the GoF/LoF of the associated gene, respectively. Since there is no experimental support for the boolean network predictions, they are marked with a question mark.

Stimulus/Perturbations	Method (In Vivo and In Vitro)	Model Response/Phenotype	References
**Cervical Cancer (HeLa Cells)**			
p53 E1 and PTEN KO in DDR	In-vitro	Senescence and apoptosis at G2/M-phase checkpoint	[61]
PTEN E1 in DDR	In-vitro	Cell cycle arrest and apoptosis at G2/M-phase checkpoint	[57]
	In-vitro	Autophagy and Apoptosis	[11]
**Breast Cancer (MCF-7 Cells)**			
p53 E1 and PTEN KO in DDR	In-vitro	Senescence and apoptosis at G2/M-phase checkpoint	[59]
PTEN E1 in DDR	In-vitro	Cell cycle arrest at the G2/M chcekpoint	[45]
	In-vitro	Cell cycle arrest and apoptosis at G2/M-phase checkpoint	[58]
	In-vitro	Autophagy and Apoptosis	[60]
**Network Predictions for In-vitro/In-vivo experiments**			
PTEN E1 together with ATM E1 in DDR	In-vivo/In-vitro	Inhibition of proliferation	?
PTEN E1 together with KO Cdc25c in DDR	In-vivo/In-vitro	Inhibition of proliferation and activation of DNA damage repair pathways	?
PTEN E1 together with NEDD4-like E3 ubiquitin-protein ligase WWP1 (WWP1) KO in DDR	In-vivo/In-vitro	Inhibition of proliferation and Induction of cell cycle arrest, senescence, autophagy and apoptosis	?

## Data Availability

Data is contained within the article or Supplementary Material.

## Supplementary Materials

The following are available online at https://www.mdpi.com/article/10.3390/cells12071085/s1, Table S1: extensive list of Boolean functions for nodes, as well as their regulators and references, Table S2: The official names of network components and biological justifications for the edges with their regulators, Data File S1: The code of the model in .sbml format.
