# Supplementary material for: Quadra-Stable Dynamics of p53 and PTEN in the DNA Damage Response
Source: Cells. 2023 Apr 4;12(7):1085. doi: 10.3390/cells12071085 (PMC10093226; doi:10.3390/cells12071085)
Supplement: Supplementary file 1 [file cells-12-01085-s001.zip › Data File S1.pdf]

Quadra-stable Dynamics of p53 and PTEN in the DNA damage response

S1 Data File

Shantanu Gupta[1], * , Pritam Kumar Panda[2], Daner A. Silveira[3], Rajeev Ahuja[2,4], and Ronaldo F. Hashimoto[1],

1 Instituto de Matemática e Estatística, Departamento de Ciência da Computação, Universidade de São Paulo, Rua do Matão 1010, 05508-090, São Paulo - SP, Brasil

2 Condensed Matter Theory Group, Materials Theory Division, Department of Physics and Astronomy, Uppsala University, Box 516, SE-751 20 Uppsala, Sweden

3 Children's Cancer Institute, Porto Alegre, RS, Brazil

4 Department of Physics, Indian Institute of Technology Ropar, Rupnagar, Punjab 140001, India

* Correspondence: shantanu.gupta@ime.usp.br; +55-11-30916135

# Code of the model in .sbml format.

```xml
<?xml version='1.0' encoding='UTF-8' standalone='no'?>
<sbml xmlns="http://www.sbml.org/sbml/level3/version1/core" layout:required="false" level="3" qual:required="true" xmlns:layout="http://www.sbml.org/sbml/level3/version1/layout/version1" version="1" xmlns:qual="http://www.sbml.org/sbml/level3/version1/qual/version1">
  <model id="model_id">
    <layout:listOfLayouts xmlns:layout="http://www.sbml.org/sbml/level3/version1/layout/version1" xmlns:xsi="http://www.w3.org/2001/XMLSchema-instance">
      <layout:layout layout:id="__layout__">
        <layout:dimensions layout:height="873" layout:width="881"/>
        <layout:listOfAdditionalGraphicalObjects>
          <layout:generalGlyph layout:id="_ly_DNA_damage" layout:reference="DNA_damage">
            <layout:boundingBox>
              <layout:position layout:x="484" layout:y="16"/>
              <layout:dimensions layout:height="45" layout:width="95"/>
            </layout:boundingBox>
          </layout:generalGlyph>
          <layout:generalGlyph layout:id="_ly_ATM_pH2AX" layout:reference="ATM_pH2AX">
            <layout:boundingBox>
              <layout:position layout:x="484" layout:y="89"/>
              <layout:dimensions layout:height="45" layout:width="80"/>
            </layout:boundingBox>
          </layout:generalGlyph>
          <layout:generalGlyph layout:id="_ly_AMPK_MAPK" layout:reference="AMPK_MAPK">
            <layout:boundingBox>
              <layout:position layout:x="170" layout:y="165"/>
              <layout:dimensions layout:height="45" layout:width="80"/>
            </layout:boundingBox>
          </layout:generalGlyph>
          <layout:generalGlyph layout:id="_ly_Mdm2" layout:reference="Mdm2">
            <layout:boundingBox>
              <layout:position layout:x="655" layout:y="226"/>
              <layout:dimensions layout:height="45" layout:width="80"/>
            </layout:boundingBox>
          </layout:generalGlyph>
          <layout:generalGlyph layout:id="_ly_p53" layout:reference="p53">
            <layout:boundingBox>
              <layout:position layout:x="485" layout:y="227"/>
              <layout:dimensions layout:height="45" layout:width="80"/>
            </layout:boundingBox>
          </layout:generalGlyph>
          <layout:generalGlyph layout:id="_ly_Wip1" layout:reference="Wip1">
            <layout:boundingBox>
              <layout:position layout:x="358" layout:y="221"/>
              <layout:dimensions layout:height="45" layout:width="80"/>
            </layout:boundingBox>
          </layout:generalGlyph>
          <layout:generalGlyph layout:id="_ly_p53_A" layout:reference="p53_A">
            <layout:boundingBox>
              <layout:position layout:x="399" layout:y="367"/>
              <layout:dimensions layout:height="45" layout:width="80"/>
            </layout:boundingBox>
          </layout:generalGlyph>
          <layout:generalGlyph layout:id="_ly_p53_K" layout:reference="p53_K">
            <layout:boundingBox>
              <layout:position layout:x="611" layout:y="367"/>
              <layout:dimensions layout:height="45" layout:width="80"/>
            </layout:boundingBox>
          </layout:generalGlyph>
```

```
<layout:generalGlyph layout:id="_ly_p21" layout:reference="p21">
  <layout:boundingBox>
    <layout:position layout:x="401" layout:y="483"/>
    <layout:dimensions layout:height="45" layout:width="80"/>
  </layout:boundingBox>
</layout:generalGlyph>
<layout:generalGlyph layout:id="_ly_p53_INP1" layout:reference="p53_INP1">
  <layout:boundingBox>
    <layout:position layout:x="316" layout:y="318"/>
    <layout:dimensions layout:height="45" layout:width="80"/>
  </layout:boundingBox>
</layout:generalGlyph>
<layout:generalGlyph layout:id="_ly_Myc" layout:reference="Myc">
  <layout:boundingBox>
    <layout:position layout:x="150" layout:y="284"/>
    <layout:dimensions layout:height="45" layout:width="80"/>
  </layout:boundingBox>
</layout:generalGlyph>
<layout:generalGlyph layout:id="_ly_Cdc25" layout:reference="Cdc25">
  <layout:boundingBox>
    <layout:position layout:x="506" layout:y="599"/>
    <layout:dimensions layout:height="45" layout:width="80"/>
  </layout:boundingBox>
</layout:generalGlyph>
<layout:generalGlyph layout:id="_ly_Cdc2_CycB" layout:reference="Cdc2_CycB">
  <layout:boundingBox>
    <layout:position layout:x="341" layout:y="601"/>
    <layout:dimensions layout:height="45" layout:width="80"/>
  </layout:boundingBox>
</layout:generalGlyph>
<layout:generalGlyph layout:id="_ly_RB" layout:reference="RB">
  <layout:boundingBox>
    <layout:position layout:x="515" layout:y="727"/>
    <layout:dimensions layout:height="45" layout:width="80"/>
  </layout:boundingBox>
</layout:generalGlyph>
<layout:generalGlyph layout:id="_ly_E2F1" layout:reference="E2F1">
  <layout:boundingBox>
    <layout:position layout:x="179" layout:y="653"/>
    <layout:dimensions layout:height="45" layout:width="80"/>
  </layout:boundingBox>
</layout:generalGlyph>
<layout:generalGlyph layout:id="_ly_Sirt_1" layout:reference="Sirt_1">
  <layout:boundingBox>
    <layout:position layout:x="291" layout:y="416"/>
    <layout:dimensions layout:height="45" layout:width="80"/>
  </layout:boundingBox>
</layout:generalGlyph>
<layout:generalGlyph layout:id="_ly_PUMA" layout:reference="PUMA">
  <layout:boundingBox>
    <layout:position layout:x="708" layout:y="733"/>
    <layout:dimensions layout:height="45" layout:width="80"/>
  </layout:boundingBox>
</layout:generalGlyph>
<layout:generalGlyph layout:id="_ly_BCL2" layout:reference="BCL2">
  <layout:boundingBox>
    <layout:position layout:x="148" layout:y="743"/>
    <layout:dimensions layout:height="45" layout:width="80"/>
  </layout:boundingBox>
</layout:generalGlyph>
<layout:generalGlyph layout:id="_ly_BAX" layout:reference="BAX">
  <layout:boundingBox>
```

```
          <layout:position layout:x="290" layout:y="828"/>
          <layout:dimensions layout:height="45" layout:width="80"/>
        </layout:boundingBox>
      </layout:generalGlyph>
      <layout:generalGlyph layout:id="_ly_Caspase" layout:reference="Caspase">
        <layout:boundingBox>
          <layout:position layout:x="543" layout:y="821"/>
          <layout:dimensions layout:height="45" layout:width="80"/>
        </layout:boundingBox>
      </layout:generalGlyph>
      <layout:generalGlyph layout:id="_ly_TUG1" layout:reference="TUG1">
        <layout:boundingBox>
          <layout:position layout:x="799" layout:y="171"/>
          <layout:dimensions layout:height="45" layout:width="80"/>
        </layout:boundingBox>
      </layout:generalGlyph>
      <layout:generalGlyph layout:id="_ly_miR221" layout:reference="miR221">
        <layout:boundingBox>
          <layout:position layout:x="798" layout:y="273"/>
          <layout:dimensions layout:height="45" layout:width="80"/>
        </layout:boundingBox>
      </layout:generalGlyph>
      <layout:generalGlyph layout:id="_ly_PTEN" layout:reference="PTEN">
        <layout:boundingBox>
          <layout:position layout:x="801" layout:y="379"/>
          <layout:dimensions layout:height="45" layout:width="80"/>
        </layout:boundingBox>
      </layout:generalGlyph>
      <layout:generalGlyph layout:id="_ly_AKT" layout:reference="AKT">
        <layout:boundingBox>
          <layout:position layout:x="801" layout:y="486"/>
          <layout:dimensions layout:height="45" layout:width="80"/>
        </layout:boundingBox>
      </layout:generalGlyph>
      <layout:generalGlyph layout:id="_ly_mTOR1" layout:reference="mTOR1">
        <layout:boundingBox>
          <layout:position layout:x="646" layout:y="603"/>
          <layout:dimensions layout:height="45" layout:width="80"/>
        </layout:boundingBox>
      </layout:generalGlyph>
      <layout:generalGlyph layout:id="_ly_mTOR2" layout:reference="mTOR2">
        <layout:boundingBox>
          <layout:position layout:x="598" layout:y="482"/>
          <layout:dimensions layout:height="45" layout:width="80"/>
        </layout:boundingBox>
      </layout:generalGlyph>
      <layout:generalGlyph layout:id="_ly_ULK1_Beclin1" layout:reference="ULK1_Beclin1">
        <layout:boundingBox>
          <layout:position layout:x="779" layout:y="816"/>
          <layout:dimensions layout:height="45" layout:width="90"/>
        </layout:boundingBox>
      </layout:generalGlyph>
      <layout:generalGlyph layout:id="_ly_WWP1" layout:reference="WWP1">
        <layout:boundingBox>
          <layout:position layout:x="645" layout:y="291"/>
          <layout:dimensions layout:height="45" layout:width="80"/>
        </layout:boundingBox>
      </layout:generalGlyph>
      <layout:generalGlyph layout:id="_ly_hTERT" layout:reference="hTERT">
        <layout:boundingBox>
          <layout:position layout:x="116" layout:y="397"/>
          <layout:dimensions layout:height="45" layout:width="80"/>
```

```
            </layout:boundingBox>
          </layout:generalGlyph>
          <layout:generalGlyph layout:id="_ly_Wee1" layout:reference="Wee1">
            <layout:boundingBox>
              <layout:position layout:x="109" layout:y="474"/>
              <layout:dimensions layout:height="45" layout:width="80"/>
            </layout:boundingBox>
          </layout:generalGlyph>
        </layout:listOfAdditionalGraphicalObjects>
      </layout:layout>
    </layout:listOfLayouts>
    <qual:listOfQualitativeSpecies xmlns:qual="http://www.sbml.org/sbml/level3/version1/qual/version1">
      <qual:qualitativeSpecies qual:maxLevel="1" qual:compartment="comp1" qual:constant="true" qual:initialLevel="1" qual:id="DNA_damage"/>
      <qual:qualitativeSpecies qual:maxLevel="1" qual:compartment="comp1" qual:constant="false" qual:id="ATM_pH2AX"/>
      <qual:qualitativeSpecies qual:maxLevel="1" qual:compartment="comp1" qual:constant="false" qual:id="AMPK_MAPK"/>
      <qual:qualitativeSpecies qual:maxLevel="1" qual:compartment="comp1" qual:constant="false" qual:id="Mdm2"/>
      <qual:qualitativeSpecies qual:maxLevel="1" qual:compartment="comp1" qual:constant="false" qual:id="p53"/>
      <qual:qualitativeSpecies qual:maxLevel="1" qual:compartment="comp1" qual:constant="false" qual:id="Wip1"/>
      <qual:qualitativeSpecies qual:maxLevel="1" qual:compartment="comp1" qual:constant="false" qual:id="p53_A"/>
      <qual:qualitativeSpecies qual:maxLevel="1" qual:compartment="comp1" qual:constant="false" qual:id="p53_K"/>
      <qual:qualitativeSpecies qual:maxLevel="1" qual:compartment="comp1" qual:constant="false" qual:id="p21"/>
      <qual:qualitativeSpecies qual:maxLevel="1" qual:compartment="comp1" qual:constant="false" qual:id="p53_INP1"/>
      <qual:qualitativeSpecies qual:maxLevel="1" qual:compartment="comp1" qual:constant="false" qual:id="Myc"/>
      <qual:qualitativeSpecies qual:maxLevel="1" qual:compartment="comp1" qual:constant="false" qual:id="Cdc25"/>
      <qual:qualitativeSpecies qual:maxLevel="1" qual:compartment="comp1" qual:constant="false" qual:id="Cdc2_CycB"/>
      <qual:qualitativeSpecies qual:maxLevel="1" qual:compartment="comp1" qual:constant="false" qual:id="RB"/>
      <qual:qualitativeSpecies qual:maxLevel="1" qual:compartment="comp1" qual:constant="false" qual:id="E2F1"/>
      <qual:qualitativeSpecies qual:maxLevel="1" qual:compartment="comp1" qual:constant="false" qual:id="Sirt_1"/>
      <qual:qualitativeSpecies qual:maxLevel="1" qual:compartment="comp1" qual:constant="false" qual:id="PUMA"/>
      <qual:qualitativeSpecies qual:maxLevel="1" qual:compartment="comp1" qual:constant="false" qual:id="BCL2"/>
      <qual:qualitativeSpecies qual:maxLevel="1" qual:compartment="comp1" qual:constant="false" qual:id="BAX"/>
      <qual:qualitativeSpecies qual:maxLevel="1" qual:compartment="comp1" qual:constant="false" qual:id="Caspase"/>
      <qual:qualitativeSpecies qual:maxLevel="1" qual:compartment="comp1" qual:constant="false" qual:id="TUG1"/>
      <qual:qualitativeSpecies qual:maxLevel="1" qual:compartment="comp1" qual:constant="false" qual:id="miR221"/>
      <qual:qualitativeSpecies qual:maxLevel="1" qual:compartment="comp1" qual:constant="false" qual:id="PTEN"/>
      <qual:qualitativeSpecies qual:maxLevel="1" qual:compartment="comp1" qual:constant="false" qual:id="AKT"/>
      <qual:qualitativeSpecies qual:maxLevel="1" qual:compartment="comp1" qual:constant="false
```

```xml
" qual:id="mTOR1"/>
      <qual:qualitativeSpecies qual:maxLevel="1" qual:compartment="comp1" qual:constant="false" qual:id="mTOR2"/>
      <qual:qualitativeSpecies qual:maxLevel="1" qual:compartment="comp1" qual:constant="false" qual:id="ULK1_Beclin1"/>
      <qual:qualitativeSpecies qual:maxLevel="1" qual:compartment="comp1" qual:constant="false" qual:id="WWP1"/>
      <qual:qualitativeSpecies qual:maxLevel="1" qual:compartment="comp1" qual:constant="false" qual:id="hTERT"/>
      <qual:qualitativeSpecies qual:maxLevel="1" qual:compartment="comp1" qual:constant="false" qual:id="Wee1"/>
    </qual:listOfQualitativeSpecies>
    <qual:listOfTransitions xmlns:qual="http://www.sbml.org/sbml/level3/version1/qual/version1">
      <qual:transition qual:id="tr_ATM_pH2AX">
        <qual:listOfInputs>
          <qual:input qual:qualitativeSpecies="DNA_damage" qual:transitionEffect="none" qual:sign="positive" qual:id="tr_ATM_pH2AX_in_0"/>
          <qual:input qual:qualitativeSpecies="Wip1" qual:transitionEffect="none" qual:sign="negative" qual:id="tr_ATM_pH2AX_in_5"/>
          <qual:input qual:qualitativeSpecies="Cdc25" qual:transitionEffect="none" qual:sign="negative" qual:id="tr_ATM_pH2AX_in_11"/>
          <qual:input qual:qualitativeSpecies="E2F1" qual:transitionEffect="none" qual:sign="positive" qual:id="tr_ATM_pH2AX_in_14"/>
        </qual:listOfInputs>
        <qual:listOfOutputs>
          <qual:output qual:qualitativeSpecies="ATM_pH2AX" qual:transitionEffect="assignmentLevel" qual:id="tr_ATM_pH2AX_out"/>
        </qual:listOfOutputs>
        <qual:listOfFunctionTerms>
          <qual:defaultTerm qual:resultLevel="0">
          </qual:defaultTerm>
          <qual:functionTerm qual:resultLevel="1">
            <math xmlns="http://www.w3.org/1998/Math/MathML">
              <apply>
                <or/>
                <apply>
                  <and/>
                  <apply>
                    <eq/>
                    <ci> DNA_damage </ci>
                    <cn type="integer"> 1 </cn>
                  </apply>
                  <apply>
                    <eq/>
                    <ci> Wip1 </ci>
                    <cn type="integer"> 0 </cn>
                  </apply>
                </apply>
                <apply>
                  <and/>
                  <apply>
                    <eq/>
                    <ci> DNA_damage </ci>
                    <cn type="integer"> 1 </cn>
                  </apply>
                  <apply>
                    <eq/>
                    <ci> Wip1 </ci>
                    <cn type="integer"> 1 </cn>
                  </apply>
                  <apply>
```

```
              <eq/>
              <ci> Cdc25 </ci>
              <cn type="integer"> 0 </cn>
            </apply>
          </apply>
          <apply>
            <and/>
            <apply>
              <eq/>
              <ci> DNA_damage </ci>
              <cn type="integer"> 1 </cn>
            </apply>
            <apply>
              <eq/>
              <ci> Wip1 </ci>
              <cn type="integer"> 1 </cn>
            </apply>
            <apply>
              <eq/>
              <ci> Cdc25 </ci>
              <cn type="integer"> 1 </cn>
            </apply>
            <apply>
              <eq/>
              <ci> E2F1 </ci>
              <cn type="integer"> 1 </cn>
            </apply>
          </apply>
        </apply>
      </math>
              </qual:functionTerm>
  </qual:listOfFunctionTerms>
</qual:transition>
<qual:transition qual:id="tr_AMPK_MAPK">
  <qual:listOfInputs>
    <qual:input qual:qualitativeSpecies="ATM_pH2AX" qual:transitionEffect="none"
         qual:sign="positive" qual:id="tr_AMPK_MAPK_in_1"/>
    <qual:input qual:qualitativeSpecies="Wip1" qual:transitionEffect="none" qual:sign="
         negative" qual:id="tr_AMPK_MAPK_in_5"/>
  </qual:listOfInputs>
  <qual:listOfOutputs>
    <qual:output qual:qualitativeSpecies="AMPK_MAPK" qual:transitionEffect="
         assignmentLevel" qual:id="tr_AMPK_MAPK_out"/>
  </qual:listOfOutputs>
  <qual:listOfFunctionTerms>
    <qual:defaultTerm qual:resultLevel="0">
    </qual:defaultTerm>
    <qual:functionTerm qual:resultLevel="1">
      <math xmlns="http://www.w3.org/1998/Math/MathML">
        <apply>
          <and/>
          <apply>
            <eq/>
            <ci> ATM_pH2AX </ci>
            <cn type="integer"> 1 </cn>
          </apply>
          <apply>
            <eq/>
            <ci> Wip1 </ci>
            <cn type="integer"> 0 </cn>
          </apply>
        </apply>
```

```xml
        </math>
            </qual:functionTerm>
      </qual:listOfFunctionTerms>
    </qual:transition>
    <qual:transition qual:id="tr_Mdm2">
      <qual:listOfInputs>
        <qual:input qual:qualitativeSpecies="ATM_pH2AX" qual:transitionEffect="none" qual:sign="negative" qual:id="tr_Mdm2_in_1"/>
        <qual:input qual:qualitativeSpecies="p53" qual:transitionEffect="none" qual:sign="positive" qual:id="tr_Mdm2_in_4"/>
        <qual:input qual:qualitativeSpecies="Wip1" qual:transitionEffect="none" qual:sign="negative" qual:id="tr_Mdm2_in_5"/>
        <qual:input qual:qualitativeSpecies="miR221" qual:transitionEffect="none" qual:sign="negative" qual:id="tr_Mdm2_in_21"/>
        <qual:input qual:qualitativeSpecies="PTEN" qual:transitionEffect="none" qual:sign="negative" qual:id="tr_Mdm2_in_22"/>
        <qual:input qual:qualitativeSpecies="AKT" qual:transitionEffect="none" qual:sign="positive" qual:id="tr_Mdm2_in_23"/>
      </qual:listOfInputs>
      <qual:listOfOutputs>
        <qual:output qual:qualitativeSpecies="Mdm2" qual:transitionEffect="assignmentLevel" qual:id="tr_Mdm2_out"/>
      </qual:listOfOutputs>
      <qual:listOfFunctionTerms>
        <qual:defaultTerm qual:resultLevel="0">
        </qual:defaultTerm>
        <qual:functionTerm qual:resultLevel="1">
          <math xmlns="http://www.w3.org/1998/Math/MathML">
            <apply>
              <or/>
              <apply>
                <and/>
                <apply>
                  <eq/>
                  <ci> ATM_pH2AX </ci>
                  <cn type="integer"> 0 </cn>
                </apply>
                <apply>
                  <eq/>
                  <ci> p53 </ci>
                  <cn type="integer"> 0 </cn>
                </apply>
                <apply>
                  <eq/>
                  <ci> Wip1 </ci>
                  <cn type="integer"> 0 </cn>
                </apply>
                <apply>
                  <eq/>
                  <ci> miR221 </ci>
                  <cn type="integer"> 0 </cn>
                </apply>
                <apply>
                  <eq/>
                  <ci> PTEN </ci>
                  <cn type="integer"> 0 </cn>
                </apply>
                <apply>
                  <eq/>
                  <ci> AKT </ci>
                  <cn type="integer"> 1 </cn>
                </apply>
```

```
          </apply>
          <apply>
            <and/>
            <apply>
              <eq/>
              <ci> ATM_pH2AX </ci>
              <cn type="integer"> 0 </cn>
            </apply>
            <apply>
              <eq/>
              <ci> p53 </ci>
              <cn type="integer"> 1 </cn>
            </apply>
            <apply>
              <eq/>
              <ci> miR221 </ci>
              <cn type="integer"> 0 </cn>
            </apply>
            <apply>
              <eq/>
              <ci> PTEN </ci>
              <cn type="integer"> 0 </cn>
            </apply>
            <apply>
              <eq/>
              <ci> AKT </ci>
              <cn type="integer"> 1 </cn>
            </apply>
          </apply>
        </apply>
      </math>
              </qual:functionTerm>
  </qual:listOfFunctionTerms>
</qual:transition>
<qual:transition qual:id="tr_p53">
  <qual:listOfInputs>
    <qual:input qual:qualitativeSpecies="ATM_pH2AX" qual:transitionEffect="none"
         qual:sign="positive" qual:id="tr_p53_in_1"/>
    <qual:input qual:qualitativeSpecies="AMPK_MAPK" qual:transitionEffect="none"
         qual:sign="positive" qual:id="tr_p53_in_2"/>
    <qual:input qual:qualitativeSpecies="Mdm2" qual:transitionEffect="none" qual:sign="
         negative" qual:id="tr_p53_in_3"/>
    <qual:input qual:qualitativeSpecies="hTERT" qual:transitionEffect="none" qual:sign="
         negative" qual:id="tr_p53_in_28"/>
  </qual:listOfInputs>
  <qual:listOfOutputs>
    <qual:output qual:qualitativeSpecies="p53" qual:transitionEffect="assignmentLevel"
         qual:id="tr_p53_out"/>
  </qual:listOfOutputs>
  <qual:listOfFunctionTerms>
    <qual:defaultTerm qual:resultLevel="0">
    </qual:defaultTerm>
    <qual:functionTerm qual:resultLevel="1">
      <math xmlns="http://www.w3.org/1998/Math/MathML">
        <apply>
          <or/>
          <apply>
            <and/>
            <apply>
              <eq/>
              <ci> ATM_pH2AX </ci>
              <cn type="integer"> 0 </cn>
```

```
                </apply>
                <apply>
                  <eq/>
                  <ci> AMPK_MAPK </ci>
                  <cn type="integer"> 1 </cn>
                </apply>
                <apply>
                  <eq/>
                  <ci> Mdm2 </ci>
                  <cn type="integer"> 0 </cn>
                </apply>
                <apply>
                  <eq/>
                  <ci> hTERT </ci>
                  <cn type="integer"> 0 </cn>
                </apply>
              </apply>
              <apply>
                <eq/>
                <ci> ATM_pH2AX </ci>
                <cn type="integer"> 1 </cn>
              </apply>
            </apply>
          </math>
                  </qual:functionTerm>
      </qual:listOfFunctionTerms>
    </qual:transition>
    <qual:transition qual:id="tr_Wip1">
      <qual:listOfInputs>
        <qual:input qual:qualitativeSpecies="p53_A" qual:transitionEffect="none" qual:sign="positive" qual:id="tr_Wip1_in_6"/>
      </qual:listOfInputs>
      <qual:listOfOutputs>
        <qual:output qual:qualitativeSpecies="Wip1" qual:transitionEffect="assignmentLevel" qual:id="tr_Wip1_out"/>
      </qual:listOfOutputs>
      <qual:listOfFunctionTerms>
        <qual:defaultTerm qual:resultLevel="0">
        </qual:defaultTerm>
        <qual:functionTerm qual:resultLevel="1">
          <math xmlns="http://www.w3.org/1998/Math/MathML">
            <apply>
              <eq/>
              <ci> p53_A </ci>
              <cn type="integer"> 1 </cn>
            </apply>
          </math>
                  </qual:functionTerm>
      </qual:listOfFunctionTerms>
    </qual:transition>
    <qual:transition qual:id="tr_p53_A">
      <qual:listOfInputs>
        <qual:input qual:qualitativeSpecies="p53" qual:transitionEffect="none" qual:sign="positive" qual:id="tr_p53_A_in_4"/>
        <qual:input qual:qualitativeSpecies="p53_K" qual:transitionEffect="none" qual:sign="negative" qual:id="tr_p53_A_in_7"/>
        <qual:input qual:qualitativeSpecies="p53_INP1" qual:transitionEffect="none" qual:sign="negative" qual:id="tr_p53_A_in_9"/>
        <qual:input qual:qualitativeSpecies="Sirt_1" qual:transitionEffect="none" qual:sign="negative" qual:id="tr_p53_A_in_15"/>
      </qual:listOfInputs>
      <qual:listOfOutputs>
```

```
      <qual:output qual:qualitativeSpecies="p53_A" qual:transitionEffect="assignmentLevel" qual:id="tr_p53_A_out"/>
    </qual:listOfOutputs>
    <qual:listOfFunctionTerms>
      <qual:defaultTerm qual:resultLevel="0">
      </qual:defaultTerm>
      <qual:functionTerm qual:resultLevel="1">
        <math xmlns="http://www.w3.org/1998/Math/MathML">
          <apply>
            <or/>
            <apply>
              <and/>
              <apply>
                <eq/>
                <ci> p53 </ci>
                <cn type="integer"> 0 </cn>
              </apply>
              <apply>
                <eq/>
                <ci> p53_K </ci>
                <cn type="integer"> 0 </cn>
              </apply>
              <apply>
                <eq/>
                <ci> p53_INP1 </ci>
                <cn type="integer"> 0 </cn>
              </apply>
              <apply>
                <eq/>
                <ci> Sirt_1 </ci>
                <cn type="integer"> 0 </cn>
              </apply>
            </apply>
            <apply>
              <and/>
              <apply>
                <eq/>
                <ci> p53 </ci>
                <cn type="integer"> 1 </cn>
              </apply>
              <apply>
                <eq/>
                <ci> p53_K </ci>
                <cn type="integer"> 0 </cn>
              </apply>
              <apply>
                <eq/>
                <ci> Sirt_1 </ci>
                <cn type="integer"> 0 </cn>
              </apply>
            </apply>
          </apply>
        </math>
                </qual:functionTerm>
    </qual:listOfFunctionTerms>
  </qual:transition>
  <qual:transition qual:id="tr_p53_K">
    <qual:listOfInputs>
      <qual:input qual:qualitativeSpecies="p53" qual:transitionEffect="none" qual:sign="positive" qual:id="tr_p53_K_in_4"/>
      <qual:input qual:qualitativeSpecies="Wip1" qual:transitionEffect="none" qual:sign="negative" qual:id="tr_p53_K_in_5"/>
```

```
          <qual:input qual:qualitativeSpecies="p53_A" qual:transitionEffect="none" qual:sign="negative" qual:id="tr_p53_K_in_6"/>
          <qual:input qual:qualitativeSpecies="Sirt_1" qual:transitionEffect="none" qual:sign="negative" qual:id="tr_p53_K_in_15"/>
        </qual:listOfInputs>
        <qual:listOfOutputs>
          <qual:output qual:qualitativeSpecies="p53_K" qual:transitionEffect="assignmentLevel" qual:id="tr_p53_K_out"/>
        </qual:listOfOutputs>
        <qual:listOfFunctionTerms>
          <qual:defaultTerm qual:resultLevel="0">
          </qual:defaultTerm>
          <qual:functionTerm qual:resultLevel="1">
            <math xmlns="http://www.w3.org/1998/Math/MathML">
              <apply>
                <or/>
                <apply>
                  <and/>
                  <apply>
                    <eq/>
                    <ci> p53 </ci>
                    <cn type="integer"> 1 </cn>
                  </apply>
                  <apply>
                    <eq/>
                    <ci> Wip1 </ci>
                    <cn type="integer"> 0 </cn>
                  </apply>
                  <apply>
                    <eq/>
                    <ci> p53_A </ci>
                    <cn type="integer"> 0 </cn>
                  </apply>
                </apply>
                <apply>
                  <and/>
                  <apply>
                    <eq/>
                    <ci> p53 </ci>
                    <cn type="integer"> 1 </cn>
                  </apply>
                  <apply>
                    <eq/>
                    <ci> Wip1 </ci>
                    <cn type="integer"> 1 </cn>
                  </apply>
                  <apply>
                    <eq/>
                    <ci> p53_A </ci>
                    <cn type="integer"> 0 </cn>
                  </apply>
                  <apply>
                    <eq/>
                    <ci> Sirt_1 </ci>
                    <cn type="integer"> 0 </cn>
                  </apply>
                </apply>
              </apply>
            </math>
                    </qual:functionTerm>
        </qual:listOfFunctionTerms>
      </qual:transition>
```

```
<qual:transition qual:id="tr_p21">
  <qual:listOfInputs>
    <qual:input qual:qualitativeSpecies="AMPK_MAPK" qual:transitionEffect="none" qual:sign="positive" qual:id="tr_p21_in_2"/>
    <qual:input qual:qualitativeSpecies="p53_A" qual:transitionEffect="none" qual:sign="positive" qual:id="tr_p21_in_6"/>
    <qual:input qual:qualitativeSpecies="Myc" qual:transitionEffect="none" qual:sign="negative" qual:id="tr_p21_in_10"/>
    <qual:input qual:qualitativeSpecies="Caspase" qual:transitionEffect="none" qual:sign="negative" qual:id="tr_p21_in_19"/>
    <qual:input qual:qualitativeSpecies="AKT" qual:transitionEffect="none" qual:sign="negative" qual:id="tr_p21_in_23"/>
  </qual:listOfInputs>
  <qual:listOfOutputs>
    <qual:output qual:qualitativeSpecies="p21" qual:transitionEffect="assignmentLevel" qual:id="tr_p21_out"/>
  </qual:listOfOutputs>
  <qual:listOfFunctionTerms>
    <qual:defaultTerm qual:resultLevel="0">
    </qual:defaultTerm>
    <qual:functionTerm qual:resultLevel="1">
      <math xmlns="http://www.w3.org/1998/Math/MathML">
        <apply>
          <or/>
          <apply>
            <and/>
            <apply>
              <eq/>
              <ci> AMPK_MAPK </ci>
              <cn type="integer"> 0 </cn>
            </apply>
            <apply>
              <eq/>
              <ci> p53_A </ci>
              <cn type="integer"> 1 </cn>
            </apply>
          </apply>
          <apply>
            <and/>
            <apply>
              <eq/>
              <ci> AMPK_MAPK </ci>
              <cn type="integer"> 1 </cn>
            </apply>
            <apply>
              <eq/>
              <ci> p53_A </ci>
              <cn type="integer"> 0 </cn>
            </apply>
            <apply>
              <eq/>
              <ci> Myc </ci>
              <cn type="integer"> 0 </cn>
            </apply>
            <apply>
              <eq/>
              <ci> Caspase </ci>
              <cn type="integer"> 0 </cn>
            </apply>
            <apply>
              <eq/>
              <ci> AKT </ci>
```

```
              <cn type="integer"> 0 </cn>
            </apply>
          </apply>
          <apply>
            <and/>
            <apply>
              <eq/>
              <ci> AMPK_MAPK </ci>
              <cn type="integer"> 1 </cn>
            </apply>
            <apply>
              <eq/>
              <ci> p53_A </ci>
              <cn type="integer"> 1 </cn>
            </apply>
          </apply>
        </apply>
      </math>
              </qual:functionTerm>
  </qual:listOfFunctionTerms>
</qual:transition>
<qual:transition qual:id="tr_p53_INP1">
  <qual:listOfInputs>
    <qual:input qual:qualitativeSpecies="p53_A" qual:transitionEffect="none" qual:sign="
        positive" qual:id="tr_p53_INP1_in_6"/>
    <qual:input qual:qualitativeSpecies="p53_K" qual:transitionEffect="none" qual:sign="
        positive" qual:id="tr_p53_INP1_in_7"/>
  </qual:listOfInputs>
  <qual:listOfOutputs>
    <qual:output qual:qualitativeSpecies="p53_INP1" qual:transitionEffect="
        assignmentLevel" qual:id="tr_p53_INP1_out"/>
  </qual:listOfOutputs>
  <qual:listOfFunctionTerms>
    <qual:defaultTerm qual:resultLevel="0">
    </qual:defaultTerm>
    <qual:functionTerm qual:resultLevel="1">
      <math xmlns="http://www.w3.org/1998/Math/MathML">
        <apply>
          <or/>
          <apply>
            <and/>
            <apply>
              <eq/>
              <ci> p53_A </ci>
              <cn type="integer"> 0 </cn>
            </apply>
            <apply>
              <eq/>
              <ci> p53_K </ci>
              <cn type="integer"> 1 </cn>
            </apply>
          </apply>
          <apply>
            <eq/>
            <ci> p53_A </ci>
            <cn type="integer"> 1 </cn>
          </apply>
        </apply>
      </math>
              </qual:functionTerm>
  </qual:listOfFunctionTerms>
</qual:transition>
```

```
<qual:transition qual:id="tr_Myc">
  <qual:listOfInputs>
    <qual:input qual:qualitativeSpecies="AMPK_MAPK" qual:transitionEffect="none" qual:sign="positive" qual:id="tr_Myc_in_2"/>
    <qual:input qual:qualitativeSpecies="p21" qual:transitionEffect="none" qual:sign="negative" qual:id="tr_Myc_in_8"/>
    <qual:input qual:qualitativeSpecies="RB" qual:transitionEffect="none" qual:sign="negative" qual:id="tr_Myc_in_13"/>
    <qual:input qual:qualitativeSpecies="E2F1" qual:transitionEffect="none" qual:sign="positive" qual:id="tr_Myc_in_14"/>
    <qual:input qual:qualitativeSpecies="AKT" qual:transitionEffect="none" qual:sign="positive" qual:id="tr_Myc_in_23"/>
  </qual:listOfInputs>
  <qual:listOfOutputs>
    <qual:output qual:qualitativeSpecies="Myc" qual:transitionEffect="assignmentLevel" qual:id="tr_Myc_out"/>
  </qual:listOfOutputs>
  <qual:listOfFunctionTerms>
    <qual:defaultTerm qual:resultLevel="0">
    </qual:defaultTerm>
    <qual:functionTerm qual:resultLevel="1">
      <math xmlns="http://www.w3.org/1998/Math/MathML">
        <apply>
          <or/>
          <apply>
            <and/>
            <apply>
              <eq/>
              <ci> AMPK_MAPK </ci>
              <cn type="integer"> 0 </cn>
            </apply>
            <apply>
              <eq/>
              <ci> p21 </ci>
              <cn type="integer"> 0 </cn>
            </apply>
            <apply>
              <eq/>
              <ci> RB </ci>
              <cn type="integer"> 0 </cn>
            </apply>
            <apply>
              <eq/>
              <ci> E2F1 </ci>
              <cn type="integer"> 0 </cn>
            </apply>
            <apply>
              <eq/>
              <ci> AKT </ci>
              <cn type="integer"> 1 </cn>
            </apply>
          </apply>
          <apply>
            <and/>
            <apply>
              <eq/>
              <ci> AMPK_MAPK </ci>
              <cn type="integer"> 0 </cn>
            </apply>
            <apply>
              <eq/>
              <ci> p21 </ci>
```

```
                          <cn type="integer"> 0 </cn>
                        </apply>
                        <apply>
                          <eq/>
                          <ci> RB </ci>
                          <cn type="integer"> 0 </cn>
                        </apply>
                        <apply>
                          <eq/>
                          <ci> E2F1 </ci>
                          <cn type="integer"> 1 </cn>
                        </apply>
                      </apply>
                      <apply>
                        <and/>
                        <apply>
                          <eq/>
                          <ci> AMPK_MAPK </ci>
                          <cn type="integer"> 1 </cn>
                        </apply>
                        <apply>
                          <eq/>
                          <ci> p21 </ci>
                          <cn type="integer"> 0 </cn>
                        </apply>
                        <apply>
                          <eq/>
                          <ci> RB </ci>
                          <cn type="integer"> 0 </cn>
                        </apply>
                      </apply>
                    </apply>
                  </math>
                          </qual:functionTerm>
              </qual:listOfFunctionTerms>
            </qual:transition>
            <qual:transition qual:id="tr_Cdc25">
              <qual:listOfInputs>
                <qual:input qual:qualitativeSpecies="ATM_pH2AX" qual:transitionEffect="none"
                     qual:sign="negative" qual:id="tr_Cdc25_in_1"/>
                <qual:input qual:qualitativeSpecies="AMPK_MAPK" qual:transitionEffect="none"
                     qual:sign="negative" qual:id="tr_Cdc25_in_2"/>
                <qual:input qual:qualitativeSpecies="PTEN" qual:transitionEffect="none" qual:sign="
                     negative" qual:id="tr_Cdc25_in_22"/>
                <qual:input qual:qualitativeSpecies="Wee1" qual:transitionEffect="none" qual:sign="
                     negative" qual:id="tr_Cdc25_in_29"/>
              </qual:listOfInputs>
              <qual:listOfOutputs>
                <qual:output qual:qualitativeSpecies="Cdc25" qual:transitionEffect="assignmentLevel"
                      qual:id="tr_Cdc25_out"/>
              </qual:listOfOutputs>
              <qual:listOfFunctionTerms>
                <qual:defaultTerm qual:resultLevel="0">
                </qual:defaultTerm>
                <qual:functionTerm qual:resultLevel="1">
                  <math xmlns="http://www.w3.org/1998/Math/MathML">
                    <apply>
                      <or/>
                      <apply>
                        <and/>
                        <apply>
                          <eq/>
```

```
                    <ci> ATM_pH2AX </ci>
                    <cn type="integer"> 0 </cn>
                  </apply>
                  <apply>
                    <eq/>
                    <ci> PTEN </ci>
                    <cn type="integer"> 0 </cn>
                  </apply>
                  <apply>
                    <eq/>
                    <ci> Weel </ci>
                    <cn type="integer"> 0 </cn>
                  </apply>
                </apply>
                <apply>
                  <and/>
                  <apply>
                    <eq/>
                    <ci> ATM_pH2AX </ci>
                    <cn type="integer"> 1 </cn>
                  </apply>
                  <apply>
                    <eq/>
                    <ci> AMPK_MAPK </ci>
                    <cn type="integer"> 0 </cn>
                  </apply>
                  <apply>
                    <eq/>
                    <ci> PTEN </ci>
                    <cn type="integer"> 0 </cn>
                  </apply>
                  <apply>
                    <eq/>
                    <ci> Weel </ci>
                    <cn type="integer"> 0 </cn>
                  </apply>
                </apply>
              </apply>
            </math>
                    </qual:functionTerm>
        </qual:listOfFunctionTerms>
      </qual:transition>
      <qual:transition qual:id="tr_Cdc2_CycB">
        <qual:listOfInputs>
          <qual:input qual:qualitativeSpecies="p21" qual:transitionEffect="none" qual:sign="
               negative" qual:id="tr_Cdc2_CycB_in_8"/>
          <qual:input qual:qualitativeSpecies="Cdc25" qual:transitionEffect="none" qual:sign="
               positive" qual:id="tr_Cdc2_CycB_in_11"/>
          <qual:input qual:qualitativeSpecies="PTEN" qual:transitionEffect="none" qual:sign="
               negative" qual:id="tr_Cdc2_CycB_in_22"/>
          <qual:input qual:qualitativeSpecies="Weel" qual:transitionEffect="none" qual:sign="
               negative" qual:id="tr_Cdc2_CycB_in_29"/>
        </qual:listOfInputs>
        <qual:listOfOutputs>
          <qual:output qual:qualitativeSpecies="Cdc2_CycB" qual:transitionEffect="
               assignmentLevel" qual:id="tr_Cdc2_CycB_out"/>
        </qual:listOfOutputs>
        <qual:listOfFunctionTerms>
          <qual:defaultTerm qual:resultLevel="0">
          </qual:defaultTerm>
          <qual:functionTerm qual:resultLevel="1">
            <math xmlns="http://www.w3.org/1998/Math/MathML">
```

```
                <apply>
                  <and/>
                  <apply>
                    <eq/>
                    <ci> p21 </ci>
                    <cn type="integer"> 0 </cn>
                  </apply>
                  <apply>
                    <eq/>
                    <ci> Cdc25 </ci>
                    <cn type="integer"> 1 </cn>
                  </apply>
                  <apply>
                    <eq/>
                    <ci> PTEN </ci>
                    <cn type="integer"> 0 </cn>
                  </apply>
                  <apply>
                    <eq/>
                    <ci> Wee1 </ci>
                    <cn type="integer"> 0 </cn>
                  </apply>
                </apply>
              </math>
                      </qual:functionTerm>
          </qual:listOfFunctionTerms>
        </qual:transition>
        <qual:transition qual:id="tr_RB">
          <qual:listOfInputs>
            <qual:input qual:qualitativeSpecies="Cdc25" qual:transitionEffect="none" qual:sign="
                negative" qual:id="tr_RB_in_11"/>
            <qual:input qual:qualitativeSpecies="Cdc2_CycB" qual:transitionEffect="none"
                qual:sign="negative" qual:id="tr_RB_in_12"/>
          </qual:listOfInputs>
          <qual:listOfOutputs>
            <qual:output qual:qualitativeSpecies="RB" qual:transitionEffect="assignmentLevel"
                qual:id="tr_RB_out"/>
          </qual:listOfOutputs>
          <qual:listOfFunctionTerms>
            <qual:defaultTerm qual:resultLevel="0">
            </qual:defaultTerm>
            <qual:functionTerm qual:resultLevel="1">
              <math xmlns="http://www.w3.org/1998/Math/MathML">
                <apply>
                  <or/>
                  <apply>
                    <eq/>
                    <ci> Cdc25 </ci>
                    <cn type="integer"> 0 </cn>
                  </apply>
                  <apply>
                    <and/>
                    <apply>
                      <eq/>
                      <ci> Cdc25 </ci>
                      <cn type="integer"> 1 </cn>
                    </apply>
                    <apply>
                      <eq/>
                      <ci> Cdc2_CycB </ci>
                      <cn type="integer"> 0 </cn>
                    </apply>
```

```
            </apply>
          </apply>
        </math>
                </qual:functionTerm>
      </qual:listOfFunctionTerms>
    </qual:transition>
    <qual:transition qual:id="tr_E2F1">
      <qual:listOfInputs>
        <qual:input qual:qualitativeSpecies="ATM_pH2AX" qual:transitionEffect="none" qual:sign="positive" qual:id="tr_E2F1_in_1"/>
        <qual:input qual:qualitativeSpecies="Myc" qual:transitionEffect="none" qual:sign="positive" qual:id="tr_E2F1_in_10"/>
        <qual:input qual:qualitativeSpecies="Cdc25" qual:transitionEffect="none" qual:sign="positive" qual:id="tr_E2F1_in_11"/>
        <qual:input qual:qualitativeSpecies="RB" qual:transitionEffect="none" qual:sign="negative" qual:id="tr_E2F1_in_13"/>
        <qual:input qual:qualitativeSpecies="Sirt_1" qual:transitionEffect="none" qual:sign="negative" qual:id="tr_E2F1_in_15"/>
        <qual:input qual:qualitativeSpecies="PTEN" qual:transitionEffect="none" qual:sign="negative" qual:id="tr_E2F1_in_22"/>
      </qual:listOfInputs>
      <qual:listOfOutputs>
        <qual:output qual:qualitativeSpecies="E2F1" qual:transitionEffect="assignmentLevel" qual:id="tr_E2F1_out"/>
      </qual:listOfOutputs>
      <qual:listOfFunctionTerms>
        <qual:defaultTerm qual:resultLevel="0">
        </qual:defaultTerm>
        <qual:functionTerm qual:resultLevel="1">
          <math xmlns="http://www.w3.org/1998/Math/MathML">
            <apply>
              <or/>
              <apply>
                <and/>
                <apply>
                  <eq/>
                  <ci> ATM_pH2AX </ci>
                  <cn type="integer"> 0 </cn>
                </apply>
                <apply>
                  <eq/>
                  <ci> Myc </ci>
                  <cn type="integer"> 0 </cn>
                </apply>
                <apply>
                  <eq/>
                  <ci> RB </ci>
                  <cn type="integer"> 0 </cn>
                </apply>
                <apply>
                  <eq/>
                  <ci> Sirt_1 </ci>
                  <cn type="integer"> 0 </cn>
                </apply>
              </apply>
              <apply>
                <and/>
                <apply>
                  <eq/>
                  <ci> ATM_pH2AX </ci>
                  <cn type="integer"> 0 </cn>
                </apply>
```

```
    <apply>
      <eq/>
      <ci> Myc </ci>
      <cn type="integer"> 0 </cn>
    </apply>
    <apply>
      <eq/>
      <ci> RB </ci>
      <cn type="integer"> 0 </cn>
    </apply>
    <apply>
      <eq/>
      <ci> Sirt_1 </ci>
      <cn type="integer"> 1 </cn>
    </apply>
    <apply>
      <eq/>
      <ci> PTEN </ci>
      <cn type="integer"> 0 </cn>
    </apply>
  </apply>
  <apply>
    <and/>
    <apply>
      <eq/>
      <ci> ATM_pH2AX </ci>
      <cn type="integer"> 0 </cn>
    </apply>
    <apply>
      <eq/>
      <ci> Myc </ci>
      <cn type="integer"> 1 </cn>
    </apply>
  </apply>
  <apply>
    <and/>
    <apply>
      <eq/>
      <ci> ATM_pH2AX </ci>
      <cn type="integer"> 1 </cn>
    </apply>
    <apply>
      <eq/>
      <ci> Myc </ci>
      <cn type="integer"> 0 </cn>
    </apply>
    <apply>
      <eq/>
      <ci> Cdc25 </ci>
      <cn type="integer"> 0 </cn>
    </apply>
    <apply>
      <eq/>
      <ci> RB </ci>
      <cn type="integer"> 0 </cn>
    </apply>
    <apply>
      <eq/>
      <ci> Sirt_1 </ci>
      <cn type="integer"> 0 </cn>
    </apply>
  </apply>
```

```
<apply>
  <and/>
  <apply>
    <eq/>
    <ci> ATM_pH2AX </ci>
    <cn type="integer"> 1 </cn>
  </apply>
  <apply>
    <eq/>
    <ci> Myc </ci>
    <cn type="integer"> 0 </cn>
  </apply>
  <apply>
    <eq/>
    <ci> Cdc25 </ci>
    <cn type="integer"> 0 </cn>
  </apply>
  <apply>
    <eq/>
    <ci> RB </ci>
    <cn type="integer"> 0 </cn>
  </apply>
  <apply>
    <eq/>
    <ci> Sirt_1 </ci>
    <cn type="integer"> 1 </cn>
  </apply>
  <apply>
    <eq/>
    <ci> PTEN </ci>
    <cn type="integer"> 0 </cn>
  </apply>
</apply>
<apply>
  <and/>
  <apply>
    <eq/>
    <ci> ATM_pH2AX </ci>
    <cn type="integer"> 1 </cn>
  </apply>
  <apply>
    <eq/>
    <ci> Myc </ci>
    <cn type="integer"> 0 </cn>
  </apply>
  <apply>
    <eq/>
    <ci> Cdc25 </ci>
    <cn type="integer"> 1 </cn>
  </apply>
  <apply>
    <eq/>
    <ci> RB </ci>
    <cn type="integer"> 0 </cn>
  </apply>
</apply>
<apply>
  <and/>
  <apply>
    <eq/>
    <ci> ATM_pH2AX </ci>
    <cn type="integer"> 1 </cn>
```

```
                </apply>
                <apply>
                  <eq/>
                  <ci> Myc </ci>
                  <cn type="integer"> 1 </cn>
                </apply>
              </apply>
            </apply>
          </math>
                  </qual:functionTerm>
      </qual:listOfFunctionTerms>
    </qual:transition>
    <qual:transition qual:id="tr_Sirt_1">
      <qual:listOfInputs>
        <qual:input qual:qualitativeSpecies="E2F1" qual:transitionEffect="none" qual:sign="
             positive" qual:id="tr_Sirt_1_in_14"/>
      </qual:listOfInputs>
      <qual:listOfOutputs>
        <qual:output qual:qualitativeSpecies="Sirt_1" qual:transitionEffect="assignmentLevel
             " qual:id="tr_Sirt_1_out"/>
      </qual:listOfOutputs>
      <qual:listOfFunctionTerms>
        <qual:defaultTerm qual:resultLevel="0">
        </qual:defaultTerm>
        <qual:functionTerm qual:resultLevel="1">
          <math xmlns="http://www.w3.org/1998/Math/MathML">
            <apply>
              <eq/>
              <ci> E2F1 </ci>
              <cn type="integer"> 1 </cn>
            </apply>
          </math>
                  </qual:functionTerm>
      </qual:listOfFunctionTerms>
    </qual:transition>
    <qual:transition qual:id="tr_PUMA">
      <qual:listOfInputs>
        <qual:input qual:qualitativeSpecies="p53_K" qual:transitionEffect="none" qual:sign="
             positive" qual:id="tr_PUMA_in_7"/>
        <qual:input qual:qualitativeSpecies="miR221" qual:transitionEffect="none" qual:sign=
             "negative" qual:id="tr_PUMA_in_21"/>
      </qual:listOfInputs>
      <qual:listOfOutputs>
        <qual:output qual:qualitativeSpecies="PUMA" qual:transitionEffect="assignmentLevel"
             qual:id="tr_PUMA_out"/>
      </qual:listOfOutputs>
      <qual:listOfFunctionTerms>
        <qual:defaultTerm qual:resultLevel="0">
        </qual:defaultTerm>
        <qual:functionTerm qual:resultLevel="1">
          <math xmlns="http://www.w3.org/1998/Math/MathML">
            <apply>
              <and/>
              <apply>
                <eq/>
                <ci> p53_K </ci>
                <cn type="integer"> 1 </cn>
              </apply>
              <apply>
                <eq/>
                <ci> miR221 </ci>
                <cn type="integer"> 0 </cn>
```

```
              </apply>
            </apply>
          </math>
                  </qual:functionTerm>
      </qual:listOfFunctionTerms>
    </qual:transition>
    <qual:transition qual:id="tr_BCL2">
      <qual:listOfInputs>
        <qual:input qual:qualitativeSpecies="p53_K" qual:transitionEffect="none" qual:sign="negative" qual:id="tr_BCL2_in_7"/>
        <qual:input qual:qualitativeSpecies="E2F1" qual:transitionEffect="none" qual:sign="positive" qual:id="tr_BCL2_in_14"/>
        <qual:input qual:qualitativeSpecies="PUMA" qual:transitionEffect="none" qual:sign="negative" qual:id="tr_BCL2_in_16"/>
      </qual:listOfInputs>
      <qual:listOfOutputs>
        <qual:output qual:qualitativeSpecies="BCL2" qual:transitionEffect="assignmentLevel" qual:id="tr_BCL2_out"/>
      </qual:listOfOutputs>
      <qual:listOfFunctionTerms>
        <qual:defaultTerm qual:resultLevel="0">
        </qual:defaultTerm>
        <qual:functionTerm qual:resultLevel="1">
          <math xmlns="http://www.w3.org/1998/Math/MathML">
            <apply>
              <and/>
              <apply>
                <eq/>
                <ci> p53_K </ci>
                <cn type="integer"> 0 </cn>
              </apply>
              <apply>
                <eq/>
                <ci> E2F1 </ci>
                <cn type="integer"> 1 </cn>
              </apply>
              <apply>
                <eq/>
                <ci> PUMA </ci>
                <cn type="integer"> 0 </cn>
              </apply>
            </apply>
          </math>
                  </qual:functionTerm>
      </qual:listOfFunctionTerms>
    </qual:transition>
    <qual:transition qual:id="tr_BAX">
      <qual:listOfInputs>
        <qual:input qual:qualitativeSpecies="p53_K" qual:transitionEffect="none" qual:sign="positive" qual:id="tr_BAX_in_7"/>
        <qual:input qual:qualitativeSpecies="BCL2" qual:transitionEffect="none" qual:sign="negative" qual:id="tr_BAX_in_17"/>
      </qual:listOfInputs>
      <qual:listOfOutputs>
        <qual:output qual:qualitativeSpecies="BAX" qual:transitionEffect="assignmentLevel" qual:id="tr_BAX_out"/>
      </qual:listOfOutputs>
      <qual:listOfFunctionTerms>
        <qual:defaultTerm qual:resultLevel="0">
        </qual:defaultTerm>
        <qual:functionTerm qual:resultLevel="1">
          <math xmlns="http://www.w3.org/1998/Math/MathML">
```

```
          <apply>
            <and/>
            <apply>
              <eq/>
              <ci> p53_K </ci>
              <cn type="integer"> 1 </cn>
            </apply>
            <apply>
              <eq/>
              <ci> BCL2 </ci>
              <cn type="integer"> 0 </cn>
            </apply>
          </apply>
        </math>
                </qual:functionTerm>
    </qual:listOfFunctionTerms>
  </qual:transition>
  <qual:transition qual:id="tr_Caspase">
    <qual:listOfInputs>
      <qual:input qual:qualitativeSpecies="p21" qual:transitionEffect="none" qual:sign="negative" qual:id="tr_Caspase_in_8"/>
      <qual:input qual:qualitativeSpecies="BCL2" qual:transitionEffect="none" qual:sign="negative" qual:id="tr_Caspase_in_17"/>
      <qual:input qual:qualitativeSpecies="BAX" qual:transitionEffect="none" qual:sign="positive" qual:id="tr_Caspase_in_18"/>
    </qual:listOfInputs>
    <qual:listOfOutputs>
      <qual:output qual:qualitativeSpecies="Caspase" qual:transitionEffect="assignmentLevel" qual:id="tr_Caspase_out"/>
    </qual:listOfOutputs>
    <qual:listOfFunctionTerms>
      <qual:defaultTerm qual:resultLevel="0">
      </qual:defaultTerm>
      <qual:functionTerm qual:resultLevel="1">
        <math xmlns="http://www.w3.org/1998/Math/MathML">
          <apply>
            <or/>
            <apply>
              <and/>
              <apply>
                <eq/>
                <ci> p21 </ci>
                <cn type="integer"> 0 </cn>
              </apply>
              <apply>
                <eq/>
                <ci> BAX </ci>
                <cn type="integer"> 1 </cn>
              </apply>
            </apply>
            <apply>
              <and/>
              <apply>
                <eq/>
                <ci> p21 </ci>
                <cn type="integer"> 1 </cn>
              </apply>
              <apply>
                <eq/>
                <ci> BCL2 </ci>
                <cn type="integer"> 0 </cn>
              </apply>
```

```
            <apply>
              <eq/>
              <ci> BAX </ci>
              <cn type="integer"> 1 </cn>
            </apply>
          </apply>
        </apply>
      </math>
              </qual:functionTerm>
  </qual:listOfFunctionTerms>
</qual:transition>
<qual:transition qual:id="tr_TUG1">
  <qual:listOfInputs>
    <qual:input qual:qualitativeSpecies="p53" qual:transitionEffect="none" qual:sign="positive" qual:id="tr_TUG1_in_4"/>
  </qual:listOfInputs>
  <qual:listOfOutputs>
    <qual:output qual:qualitativeSpecies="TUG1" qual:transitionEffect="assignmentLevel" qual:id="tr_TUG1_out"/>
  </qual:listOfOutputs>
  <qual:listOfFunctionTerms>
    <qual:defaultTerm qual:resultLevel="0">
    </qual:defaultTerm>
    <qual:functionTerm qual:resultLevel="1">
      <math xmlns="http://www.w3.org/1998/Math/MathML">
        <apply>
          <eq/>
          <ci> p53 </ci>
          <cn type="integer"> 1 </cn>
        </apply>
      </math>
              </qual:functionTerm>
  </qual:listOfFunctionTerms>
</qual:transition>
<qual:transition qual:id="tr_miR221">
  <qual:listOfInputs>
    <qual:input qual:qualitativeSpecies="E2F1" qual:transitionEffect="none" qual:sign="positive" qual:id="tr_miR221_in_14"/>
    <qual:input qual:qualitativeSpecies="TUG1" qual:transitionEffect="none" qual:sign="negative" qual:id="tr_miR221_in_20"/>
  </qual:listOfInputs>
  <qual:listOfOutputs>
    <qual:output qual:qualitativeSpecies="miR221" qual:transitionEffect="assignmentLevel" qual:id="tr_miR221_out"/>
  </qual:listOfOutputs>
  <qual:listOfFunctionTerms>
    <qual:defaultTerm qual:resultLevel="0">
    </qual:defaultTerm>
    <qual:functionTerm qual:resultLevel="1">
      <math xmlns="http://www.w3.org/1998/Math/MathML">
        <apply>
          <or/>
          <apply>
            <and/>
            <apply>
              <eq/>
              <ci> E2F1 </ci>
              <cn type="integer"> 0 </cn>
            </apply>
            <apply>
              <eq/>
              <ci> TUG1 </ci>
```

```
              <cn type="integer"> 0 </cn>
            </apply>
          </apply>
          <apply>
            <eq/>
            <ci> E2F1 </ci>
            <cn type="integer"> 1 </cn>
          </apply>
        </apply>
      </math>
              </qual:functionTerm>
  </qual:listOfFunctionTerms>
</qual:transition>
<qual:transition qual:id="tr_PTEN">
  <qual:listOfInputs>
    <qual:input qual:qualitativeSpecies="ATM_pH2AX" qual:transitionEffect="none" qual:sign="positive" qual:id="tr_PTEN_in_1"/>
    <qual:input qual:qualitativeSpecies="p53_K" qual:transitionEffect="none" qual:sign="positive" qual:id="tr_PTEN_in_7"/>
    <qual:input qual:qualitativeSpecies="miR221" qual:transitionEffect="none" qual:sign="negative" qual:id="tr_PTEN_in_21"/>
    <qual:input qual:qualitativeSpecies="WWP1" qual:transitionEffect="none" qual:sign="negative" qual:id="tr_PTEN_in_27"/>
  </qual:listOfInputs>
  <qual:listOfOutputs>
    <qual:output qual:qualitativeSpecies="PTEN" qual:transitionEffect="assignmentLevel" qual:id="tr_PTEN_out"/>
  </qual:listOfOutputs>
  <qual:listOfFunctionTerms>
    <qual:defaultTerm qual:resultLevel="0">
    </qual:defaultTerm>
    <qual:functionTerm qual:resultLevel="1">
      <math xmlns="http://www.w3.org/1998/Math/MathML">
        <apply>
          <or/>
          <apply>
            <and/>
            <apply>
              <eq/>
              <ci> ATM_pH2AX </ci>
              <cn type="integer"> 0 </cn>
            </apply>
            <apply>
              <eq/>
              <ci> p53_K </ci>
              <cn type="integer"> 1 </cn>
            </apply>
            <apply>
              <eq/>
              <ci> miR221 </ci>
              <cn type="integer"> 0 </cn>
            </apply>
            <apply>
              <eq/>
              <ci> WWP1 </ci>
              <cn type="integer"> 0 </cn>
            </apply>
          </apply>
          <apply>
            <and/>
            <apply>
              <eq/>
```

```xml
                <ci> ATM_pH2AX </ci>
                <cn type="integer"> 1 </cn>
              </apply>
              <apply>
                <eq/>
                <ci> miR221 </ci>
                <cn type="integer"> 0 </cn>
              </apply>
              <apply>
                <eq/>
                <ci> WWP1 </ci>
                <cn type="integer"> 0 </cn>
              </apply>
            </apply>
          </apply>
        </math>
                </qual:functionTerm>
    </qual:listOfFunctionTerms>
  </qual:transition>
  <qual:transition qual:id="tr_AKT">
    <qual:listOfInputs>
      <qual:input qual:qualitativeSpecies="PTEN" qual:transitionEffect="none" qual:sign="negative" qual:id="tr_AKT_in_22"/>
      <qual:input qual:qualitativeSpecies="mTOR2" qual:transitionEffect="none" qual:sign="positive" qual:id="tr_AKT_in_25"/>
    </qual:listOfInputs>
    <qual:listOfOutputs>
      <qual:output qual:qualitativeSpecies="AKT" qual:transitionEffect="assignmentLevel" qual:id="tr_AKT_out"/>
    </qual:listOfOutputs>
    <qual:listOfFunctionTerms>
      <qual:defaultTerm qual:resultLevel="0">
      </qual:defaultTerm>
      <qual:functionTerm qual:resultLevel="1">
        <math xmlns="http://www.w3.org/1998/Math/MathML">
          <apply>
            <or/>
            <apply>
              <eq/>
              <ci> PTEN </ci>
              <cn type="integer"> 0 </cn>
            </apply>
            <apply>
              <and/>
              <apply>
                <eq/>
                <ci> PTEN </ci>
                <cn type="integer"> 1 </cn>
              </apply>
              <apply>
                <eq/>
                <ci> mTOR2 </ci>
                <cn type="integer"> 1 </cn>
              </apply>
            </apply>
          </apply>
        </math>
                </qual:functionTerm>
    </qual:listOfFunctionTerms>
  </qual:transition>
  <qual:transition qual:id="tr_mTOR1">
    <qual:listOfInputs>
```

```
        <qual:input qual:qualitativeSpecies="AMPK_MAPK" qual:transitionEffect="none" qual:sign="negative" qual:id="tr_mTOR1_in_2"/>
        <qual:input qual:qualitativeSpecies="AKT" qual:transitionEffect="none" qual:sign="positive" qual:id="tr_mTOR1_in_23"/>
        <qual:input qual:qualitativeSpecies="ULK1_Beclin1" qual:transitionEffect="none" qual:sign="negative" qual:id="tr_mTOR1_in_26"/>
      </qual:listOfInputs>
      <qual:listOfOutputs>
        <qual:output qual:qualitativeSpecies="mTOR1" qual:transitionEffect="assignmentLevel" qual:id="tr_mTOR1_out"/>
      </qual:listOfOutputs>
      <qual:listOfFunctionTerms>
        <qual:defaultTerm qual:resultLevel="0">
        </qual:defaultTerm>
        <qual:functionTerm qual:resultLevel="1">
          <math xmlns="http://www.w3.org/1998/Math/MathML">
            <apply>
              <or/>
              <apply>
                <and/>
                <apply>
                  <eq/>
                  <ci> AMPK_MAPK </ci>
                  <cn type="integer"> 0 </cn>
                </apply>
                <apply>
                  <eq/>
                  <ci> AKT </ci>
                  <cn type="integer"> 1 </cn>
                </apply>
              </apply>
              <apply>
                <and/>
                <apply>
                  <eq/>
                  <ci> AMPK_MAPK </ci>
                  <cn type="integer"> 1 </cn>
                </apply>
                <apply>
                  <eq/>
                  <ci> AKT </ci>
                  <cn type="integer"> 1 </cn>
                </apply>
                <apply>
                  <eq/>
                  <ci> ULK1_Beclin1 </ci>
                  <cn type="integer"> 0 </cn>
                </apply>
              </apply>
            </apply>
          </math>
                  </qual:functionTerm>
      </qual:listOfFunctionTerms>
    </qual:transition>
    <qual:transition qual:id="tr_mTOR2">
      <qual:listOfInputs>
        <qual:input qual:qualitativeSpecies="AMPK_MAPK" qual:transitionEffect="none" qual:sign="negative" qual:id="tr_mTOR2_in_2"/>
        <qual:input qual:qualitativeSpecies="Sirt_1" qual:transitionEffect="none" qual:sign="negative" qual:id="tr_mTOR2_in_15"/>
        <qual:input qual:qualitativeSpecies="PTEN" qual:transitionEffect="none" qual:sign="negative" qual:id="tr_mTOR2_in_22"/>
```

```
          <qual:input qual:qualitativeSpecies="AKT" qual:transitionEffect="none" qual:sign="
               positive" qual:id="tr_mTOR2_in_23"/>
          <qual:input qual:qualitativeSpecies="mTOR1" qual:transitionEffect="none" qual:sign="
               negative" qual:id="tr_mTOR2_in_24"/>
          <qual:input qual:qualitativeSpecies="hTERT" qual:transitionEffect="none" qual:sign="
               negative" qual:id="tr_mTOR2_in_28"/>
        </qual:listOfInputs>
        <qual:listOfOutputs>
          <qual:output qual:qualitativeSpecies="mTOR2" qual:transitionEffect="assignmentLevel"
                qual:id="tr_mTOR2_out"/>
        </qual:listOfOutputs>
        <qual:listOfFunctionTerms>
          <qual:defaultTerm qual:resultLevel="0">
          </qual:defaultTerm>
          <qual:functionTerm qual:resultLevel="1">
            <math xmlns="http://www.w3.org/1998/Math/MathML">
              <apply>
                <or/>
                <apply>
                  <and/>
                  <apply>
                    <eq/>
                    <ci> AMPK_MAPK </ci>
                    <cn type="integer"> 0 </cn>
                  </apply>
                  <apply>
                    <eq/>
                    <ci> PTEN </ci>
                    <cn type="integer"> 0 </cn>
                  </apply>
                </apply>
                <apply>
                  <and/>
                  <apply>
                    <eq/>
                    <ci> AMPK_MAPK </ci>
                    <cn type="integer"> 0 </cn>
                  </apply>
                  <apply>
                    <eq/>
                    <ci> PTEN </ci>
                    <cn type="integer"> 1 </cn>
                  </apply>
                  <apply>
                    <eq/>
                    <ci> AKT </ci>
                    <cn type="integer"> 1 </cn>
                  </apply>
                </apply>
                <apply>
                  <and/>
                  <apply>
                    <eq/>
                    <ci> AMPK_MAPK </ci>
                    <cn type="integer"> 1 </cn>
                  </apply>
                  <apply>
                    <eq/>
                    <ci> Sirt_1 </ci>
                    <cn type="integer"> 0 </cn>
                  </apply>
                  <apply>
```

```
    <eq/>
    <ci> PTEN </ci>
    <cn type="integer"> 0 </cn>
  </apply>
</apply>
<apply>
  <and/>
  <apply>
    <eq/>
    <ci> AMPK_MAPK </ci>
    <cn type="integer"> 1 </cn>
  </apply>
  <apply>
    <eq/>
    <ci> Sirt_1 </ci>
    <cn type="integer"> 0 </cn>
  </apply>
  <apply>
    <eq/>
    <ci> PTEN </ci>
    <cn type="integer"> 1 </cn>
  </apply>
  <apply>
    <eq/>
    <ci> AKT </ci>
    <cn type="integer"> 1 </cn>
  </apply>
</apply>
<apply>
  <and/>
  <apply>
    <eq/>
    <ci> AMPK_MAPK </ci>
    <cn type="integer"> 1 </cn>
  </apply>
  <apply>
    <eq/>
    <ci> Sirt_1 </ci>
    <cn type="integer"> 1 </cn>
  </apply>
  <apply>
    <eq/>
    <ci> PTEN </ci>
    <cn type="integer"> 0 </cn>
  </apply>
  <apply>
    <eq/>
    <ci> AKT </ci>
    <cn type="integer"> 0 </cn>
  </apply>
  <apply>
    <eq/>
    <ci> mTOR1 </ci>
    <cn type="integer"> 0 </cn>
  </apply>
</apply>
<apply>
  <and/>
  <apply>
    <eq/>
    <ci> AMPK_MAPK </ci>
    <cn type="integer"> 1 </cn>
```

```
      </apply>
      <apply>
        <eq/>
        <ci> Sirt_1 </ci>
        <cn type="integer"> 1 </cn>
      </apply>
      <apply>
        <eq/>
        <ci> PTEN </ci>
        <cn type="integer"> 0 </cn>
      </apply>
      <apply>
        <eq/>
        <ci> AKT </ci>
        <cn type="integer"> 0 </cn>
      </apply>
      <apply>
        <eq/>
        <ci> mTOR1 </ci>
        <cn type="integer"> 1 </cn>
      </apply>
      <apply>
        <eq/>
        <ci> hTERT </ci>
        <cn type="integer"> 0 </cn>
      </apply>
    </apply>
    <apply>
      <and/>
      <apply>
        <eq/>
        <ci> AMPK_MAPK </ci>
        <cn type="integer"> 1 </cn>
      </apply>
      <apply>
        <eq/>
        <ci> Sirt_1 </ci>
        <cn type="integer"> 1 </cn>
      </apply>
      <apply>
        <eq/>
        <ci> PTEN </ci>
        <cn type="integer"> 0 </cn>
      </apply>
      <apply>
        <eq/>
        <ci> AKT </ci>
        <cn type="integer"> 1 </cn>
      </apply>
    </apply>
    <apply>
      <and/>
      <apply>
        <eq/>
        <ci> AMPK_MAPK </ci>
        <cn type="integer"> 1 </cn>
      </apply>
      <apply>
        <eq/>
        <ci> Sirt_1 </ci>
        <cn type="integer"> 1 </cn>
      </apply>
```

```
            <apply>
              <eq/>
              <ci> PTEN </ci>
              <cn type="integer"> 1 </cn>
            </apply>
            <apply>
              <eq/>
              <ci> AKT </ci>
              <cn type="integer"> 1 </cn>
            </apply>
          </apply>
        </apply>
      </math>
              </qual:functionTerm>
  </qual:listOfFunctionTerms>
</qual:transition>
<qual:transition qual:id="tr_ULK1_Beclin1">
  <qual:listOfInputs>
    <qual:input qual:qualitativeSpecies="AMPK_MAPK" qual:transitionEffect="none" qual:sign="positive" qual:id="tr_ULK1_Beclin1_in_2"/>
    <qual:input qual:qualitativeSpecies="mTOR1" qual:transitionEffect="none" qual:sign="negative" qual:id="tr_ULK1_Beclin1_in_24"/>
    <qual:input qual:qualitativeSpecies="mTOR2" qual:transitionEffect="none" qual:sign="negative" qual:id="tr_ULK1_Beclin1_in_25"/>
  </qual:listOfInputs>
  <qual:listOfOutputs>
    <qual:output qual:qualitativeSpecies="ULK1_Beclin1" qual:transitionEffect="assignmentLevel" qual:id="tr_ULK1_Beclin1_out"/>
  </qual:listOfOutputs>
  <qual:listOfFunctionTerms>
    <qual:defaultTerm qual:resultLevel="0">
    </qual:defaultTerm>
    <qual:functionTerm qual:resultLevel="1">
      <math xmlns="http://www.w3.org/1998/Math/MathML">
        <apply>
          <and/>
          <apply>
            <eq/>
            <ci> AMPK_MAPK </ci>
            <cn type="integer"> 1 </cn>
          </apply>
          <apply>
            <eq/>
            <ci> mTOR1 </ci>
            <cn type="integer"> 0 </cn>
          </apply>
          <apply>
            <eq/>
            <ci> mTOR2 </ci>
            <cn type="integer"> 0 </cn>
          </apply>
        </apply>
      </math>
              </qual:functionTerm>
  </qual:listOfFunctionTerms>
</qual:transition>
<qual:transition qual:id="tr_WWP1">
  <qual:listOfInputs>
    <qual:input qual:qualitativeSpecies="Myc" qual:transitionEffect="none" qual:sign="positive" qual:id="tr_WWP1_in_10"/>
  </qual:listOfInputs>
  <qual:listOfOutputs>
```

```
        <qual:output qual:qualitativeSpecies="WWP1" qual:transitionEffect="assignmentLevel"
             qual:id="tr_WWP1_out"/>
      </qual:listOfOutputs>
      <qual:listOfFunctionTerms>
        <qual:defaultTerm qual:resultLevel="0">
        </qual:defaultTerm>
        <qual:functionTerm qual:resultLevel="1">
          <math xmlns="http://www.w3.org/1998/Math/MathML">
            <apply>
              <eq/>
              <ci> Myc </ci>
              <cn type="integer"> 1 </cn>
            </apply>
          </math>
                  </qual:functionTerm>
      </qual:listOfFunctionTerms>
    </qual:transition>
    <qual:transition qual:id="tr_hTERT">
      <qual:listOfInputs>
        <qual:input qual:qualitativeSpecies="p53" qual:transitionEffect="none" qual:sign="
             negative" qual:id="tr_hTERT_in_4"/>
        <qual:input qual:qualitativeSpecies="Myc" qual:transitionEffect="none" qual:sign="
             positive" qual:id="tr_hTERT_in_10"/>
        <qual:input qual:qualitativeSpecies="PTEN" qual:transitionEffect="none" qual:sign="
             negative" qual:id="tr_hTERT_in_22"/>
        <qual:input qual:qualitativeSpecies="AKT" qual:transitionEffect="none" qual:sign="
             positive" qual:id="tr_hTERT_in_23"/>
      </qual:listOfInputs>
      <qual:listOfOutputs>
        <qual:output qual:qualitativeSpecies="hTERT" qual:transitionEffect="assignmentLevel"
              qual:id="tr_hTERT_out"/>
      </qual:listOfOutputs>
      <qual:listOfFunctionTerms>
        <qual:defaultTerm qual:resultLevel="0">
        </qual:defaultTerm>
        <qual:functionTerm qual:resultLevel="1">
          <math xmlns="http://www.w3.org/1998/Math/MathML">
            <apply>
              <or/>
              <apply>
                <and/>
                <apply>
                  <eq/>
                  <ci> p53 </ci>
                  <cn type="integer"> 0 </cn>
                </apply>
                <apply>
                  <eq/>
                  <ci> Myc </ci>
                  <cn type="integer"> 0 </cn>
                </apply>
                <apply>
                  <eq/>
                  <ci> PTEN </ci>
                  <cn type="integer"> 0 </cn>
                </apply>
                <apply>
                  <eq/>
                  <ci> AKT </ci>
                  <cn type="integer"> 1 </cn>
                </apply>
              </apply>
```

```
                <apply>
                  <and/>
                  <apply>
                    <eq/>
                    <ci> p53 </ci>
                    <cn type="integer"> 0 </cn>
                  </apply>
                  <apply>
                    <eq/>
                    <ci> Myc </ci>
                    <cn type="integer"> 1 </cn>
                  </apply>
                  <apply>
                    <eq/>
                    <ci> PTEN </ci>
                    <cn type="integer"> 0 </cn>
                  </apply>
                </apply>
              </apply>
            </math>
                    </qual:functionTerm>
        </qual:listOfFunctionTerms>
      </qual:transition>
      <qual:transition qual:id="tr_Wee1">
        <qual:listOfInputs>
          <qual:input qual:qualitativeSpecies="AKT" qual:transitionEffect="none" qual:sign="
              negative" qual:id="tr_Wee1_in_23"/>
        </qual:listOfInputs>
        <qual:listOfOutputs>
          <qual:output qual:qualitativeSpecies="Wee1" qual:transitionEffect="assignmentLevel"
              qual:id="tr_Wee1_out"/>
        </qual:listOfOutputs>
        <qual:listOfFunctionTerms>
          <qual:defaultTerm qual:resultLevel="0">
          </qual:defaultTerm>
          <qual:functionTerm qual:resultLevel="1">
            <math xmlns="http://www.w3.org/1998/Math/MathML">
              <apply>
                <eq/>
                <ci> AKT </ci>
                <cn type="integer"> 0 </cn>
              </apply>
            </math>
                    </qual:functionTerm>
        </qual:listOfFunctionTerms>
      </qual:transition>
    </qual:listOfTransitions>
    <listOfCompartments>
      <compartment constant="true" id="comp1"/>
    </listOfCompartments>
  </model>
</sbml>
```
