# Supplementary material for: Quadra-Stable Dynamics of p53 and PTEN in the DNA Damage Response
Source: Cells. 2023 Apr 4;12(7):1085. doi: 10.3390/cells12071085 (PMC10093226; doi:10.3390/cells12071085)
Supplement: Supplementary file 1 [file cells-12-01085-s001.zip › Table S1.pdf]

Quadra-stable Dynamics of p53 and PTEN in the DNA damage response

Table S1

Shantanu Gupta [1, *], Pritam Kumar Panda [2,], Daner A. Silveira [3,], Rajeev Ahuja [2,4], and Ronaldo F. Hashimoto [1,]

[1] Instituto de Matemática e Estatística, Departamento de Ciência da Computação, Universidade de São Paulo, Rua do Matão 1010, 05508-090, São Paulo - SP, Brasil
[2] Condensed Matter Theory Group, Materials Theory Division, Department of Physics and Astronomy, Uppsala University, Box 516, SE-751 20 Uppsala, Sweden
[3] Children's Cancer Institute, Porto Alegre, RS, Brazil
[4] Department of Physics, Indian Institute of Technology Ropar, Rupnagar, Punjab 140001, India

Correspondence: shantanu.gupta@ime.usp.br; +55-11-30916135

Table S1: Each node's Boolean control function logically explains the combined impact of the node regulators. The regulatory functions of the nodes are included here along with their regulators as well as supporting references. The complete name of the network elements and the biological explanation for the edges coordinating with each node are provided in Table S2.

**Node**	**Regulatory Functions**	**References**
DNA damage	When DNA damage present in cancer cells	-
ATM-pH2AX	DNA damage AND (NOT Wip1 OR NOT Cdc25 OR E2F1)	[1, 2, 3, 4]
AMPK/MAPK	ATM-pH2AX AND NOT Wip1	[5, 6, 7]
Mdm2	(NOT Wip1 OR p53) AND NOT ATM-pH2AX AND NOT miR221 AND NOT PTEN AND AKT	[8, 9, 10, 11, 12]
p53	ATM-pH2AX OR (NOT Mdm2 AND AMPK-MAPK AND NOT hTERT)	[1, 13, 14, 15, 16]
p53-A	NOT Sirt1 AND NOT p53-K AND (p53 OR NOT p53INP1)	[17, 18]
p53-INP1	p53-K OR p53-A	[18]
p53-K	NOT p53-A AND (NOT Sirt1 OR NOT Wip1) AND p53	[18, 17, 8]
p21	p53-A OR (NOT Myc AND NOT AKT AND NOT Caspase AND AMPK-MAPK)	[19, 20, 21, 22, 23, 24]
Wip1	p53-A	[25]
RB	NOT (Cdc25 AND Cdc2-CycB)	[26, 27]
Myc	(E2F1 OR AMPK-MAPK OR AKT) AND NOT RB AND NOT p21	[28, 29, 30, 31, 32, 33]
WWP1	Myc	[34]
hTERT	(AKT OR Myc) AND NOT p53 AND NOT PTEN	[35, 36, 16, 37]
E2F1	(NOT RB AND ((Cdc25 AND ATM-pH2AX) OR NOT Sirt1 OR NOT PTEN)) OR Myc	[38, 39, 40, 41, 42, 43]
Cdc25	(NOT ATM-pH2AX OR NOT AMPK-MAPK) AND NOT Wee1 AND NOT PTEN	[44, 45, 5, 46, 3]
Sirt1	E2F1	[42]
Cdc2-CycB	Cdc25 AND NOT Wee1 AND NOT PTEN AND NOT p21	[47, 48, 49, 21]
Wee1	NOT AKT	[46]
TUG1	p53	[50]
miR-221	NOT TUG1 OR E2F1	[37, 51]
PTEN	NOT miR-221 AND (ATM-pH2AX OR p53-K) AND NOT WWP1	[37, 52, 53, 34]
AKT	NOT PTEN OR mTOR2	[37, 54]
mTORC1	AKT AND NOT (ULK1/Beclin1 AND AMPK-MAPK)	[55, 56, 57, 58]
mTORC2	AKT OR (NOT PTEN AND (NOT mTOR1 OR NOT Sirt1 OR NOT hTERT OR NOT AMPK-MAPK))	[54, 59, 60, 61, 62, 57, 58]
ULK1/Beclin1	NOT mTOR2 AND NOT mTOR1 AND AMPK-MAPK	[63, 64, 57]
BCL2	NOT PUMA AND E2F1 AND NOT p53-K	[65, 66, 67]
PUMA	p53-K AND NOT miR-221	[68, 69]
BAX	NOT BCL2 AND p53-K	[70, 71]
Caspase	NOT (BCL2 AND p21) AND BAX	[72, 73, 74]

References

[1] Fernandez-Capetillo O, Chen HT, Celeste A, Ward I, Romanienko PJ, Morales JC, et al. DNA damage-induced G2–M checkpoint activation by histone H2AX and 53BP1. Nature cell biology. 2002;4(12):993-7.

[2] Tatewaki N, Konishi T, Nakajima Y, Nishida M, Saito M, Eitsuka T, et al. Squalene inhibits ATM-dependent signaling in γIR-induced DNA damage response through induction of Wip1 phosphatase. PloS one. 2016;11(1):e0147570.

[3] Zhang R, Zhu L, Zhang L, Xu A, Li Z, Xu Y, et al. PTEN enhances G2/M arrest in etoposide-treated MCF-7 cells through activation of the ATM pathway. Oncology reports. 2016;35(5):2707-14.

[4] Lin WC, Lin FT, Nevins JR. Selective induction of E2F1 in response to DNA damage, mediated by ATM-dependent phosphorylation. Genes & development. 2001;15(14):1833-44.

[5] Zhang M, Qu J, Gao Z, Qi Q, Yin H, Zhu L, et al. Timosaponin AIII induces G2/M arrest and apoptosis in breast cancer by activating the ATM/Chk2 and p38 MAPK signaling pathways. Frontiers in Pharmacology. 2021;11:601468.

[6] Zou Y, Wang Q, Li B, Xie B, Wang W. Temozolomide induces autophagy via ATM-AMPK-ULK1 pathways in glioma. Molecular medicine reports. 2014;10(1):411-6.

[7] Takekawa M, Adachi M, Nakahata A, Nakayama I, Itoh F, Tsukuda H, et al. p53-inducible wip1 phosphatase mediates a negative feedback regulation of p38 MAPK-p53 signaling in response to UV radiation. The EMBO journal. 2000;19(23):6517-26.

[8] Lu X, Ma O, Nguyen TA, Jones SN, Oren M, Donehower LA. The Wip1 Phosphatase acts as a gatekeeper in the p53-Mdm2 autoregulatory loop. Cancer cell. 2007;12(4):342-54.

[9] Meulmeester E, Pereg Y, Shiloh Y, Jochemsen AG. ATM-mediated phosphorylations inhibit Mdmx/Mdm2 stabilization by HAUSP in favor of p53 activation. Cell Cycle. 2005;4(9):1166-70.

[10] Ni L, Xu J, Zhao F, Dai X, Tao J, Pan J, et al. MiR-221-3p-mediated downregulation of MDM2 reverses the paclitaxel resistance of non-small cell lung cancer in vitro and in vivo. European journal of pharmacology. 2021;899:174054.

[11] Mayo LD, Dixon JE, Durden DL, Tonks NK, Donner DB. PTEN protects p53 from Mdm2 and sensitizes cancer cells to chemotherapy. Journal of Biological Chemistry. 2002;277(7):5484-9.

[12] Mayo LD, Donner DB. A phosphatidylinositol 3-kinase/Akt pathway promotes translocation of Mdm2 from the cytoplasm to the nucleus. Proceedings of the National Academy of Sciences. 2001;98(20):11598-603.

[13] Chène P. Inhibiting the p53–MDM2 interaction: an important target for cancer therapy. Nature reviews cancer. 2003;3(2):102-9.

[14] De S, Campbell C, Venkitaraman AR, Esposito A. Pulsatile MAPK signaling modulates p53 activity to control cell fate decisions at the G2 checkpoint for DNA damage. Cell reports. 2020;30(7):2083-93.

[15] He P, Li Z, Xu F, Ru G, Huang Y, Lin E, et al. AMPK activity contributes to G2 arrest and DNA damage decrease via p53/p21 pathways in oxidatively damaged mouse zygotes. Frontiers in Cell and Developmental Biology. 2020;8:539485.

[16] Chen RJ, Wu PH, Ho CT, Way TD, Pan MH, Chen HM, et al. P53-dependent downregulation of hTERT protein expression and telomerase activity induces senescence in lung cancer cells as a result of pterostilbene treatment. Cell death & disease. 2017;8(8):e2985-5.

[17] Yi J, Luo J. SIRT1 and p53, effect on cancer, senescence and beyond. Biochimica et Biophysica Acta (BBA)-Proteins and Proteomics. 2010;1804(8):1684-9.

[18] Zhang XP, Liu F, Wang W. Two-phase dynamics of p53 in the DNA damage response. Proceedings of the National Academy of Sciences. 2011;108(22):8990-5.

[19] Engeland K. Cell cycle regulation: p53-p21-RB signaling. Cell Death & Differentiation. 2022;29(5):946-60.

[20] Zhang J, Song N, Zang D, Yu J, Li J, Di W, et al. c-Myc promotes tumor proliferation and anti-apoptosis by repressing p21 in rhabdomyosarcomas. Molecular Medicine Reports. 2017;16(4):4089-94.

[21] Park JK, Jung HY, Park SH, Kang SY, Yi MR, Um HD, et al. Combination of PTEN and γ-ionizing radiation enhances cell death and G2/M arrest through regulation of AKT activity and p21 induction in non–small-cell lung cancer cells. International Journal of Radiation Oncology* Biology* Physics. 2008;70(5):1552-60.

[22] Zhang Y, Fujita N, Tsuruo T. Caspase-mediated cleavage of p21Waf1/Cip1 converts cancer cells from growth arrest to undergoing apoptosis. Oncogene. 1999;18(5):1131-8.

[23] Kong D, Dagon Y, Campbell JN, Guo Y, Yang Z, Yi X, et al. A postsynaptic AMPK→ p21-activated kinase pathway drives fasting-induced synaptic plasticity in AgRP neurons. Neuron. 2016;91(1):25-33.

[24] Ostrovsky O, Bengal E. The mitogen-activated protein kinase cascade promotes myoblast cell survival by stabilizing the cyclin-dependent kinase inhibitor, p21WAF1 protein. Journal of Biological Chemistry. 2003;278(23):21221-31.

[25] Fiscella M, Zhang H, Fan S, Sakaguchi K, Shen S, Mercer WE, et al. Wip1, a novel human protein phosphatase that is induced in response to ionizing radiation in a p53-dependent manner. Proceedings of the National Academy of Sciences. 1997;94(12):6048-53.

[26] Schade AE, Fischer M, DeCaprio JA. RB, p130 and p107 differentially repress G1/S and G2/M genes after p53 activation. Nucleic Acids Research. 2019;47(21):11197-208.

[27] Naderi S, Hunton IC, Wang JY. Radiation dose-dependent maintenance of G2 arrest requires retinoblastoma protein. Cell Cycle. 2002;1(3):192-9.

[28] Leung J, Ehmann G, Giangrande P, Nevins J. A role for Myc in facilitating transcription activation by E2F1. Oncogene. 2008;27(30):4172-9.

[29] Kfoury A, Armaro M, Collodet C, Sordet-Dessimoz J, Giner MP, Christen S, et al. AMPK promotes survival of c-Myc-positive melanoma cells by suppressing oxidative stress. The EMBO journal. 2018;37(5):e97673.

[30] Pathria G, Verma S, Yin J, Scott DA, Ronai ZA. MAPK signaling regulates c-MYC for melanoma cell adaptation to asparagine restriction. EMBO reports. 2021;22(3):e51436.

[31] Bouchard C, Marquardt J, Bras A, Medema RH, Eilers M. Myc-induced proliferation and transformation require Akt-mediated phosphorylation of FoxO proteins. The EMBO journal. 2004;23(14):2830-40.

[32] Zhao X, Day ML. RB activation and repression of C-MYC transcription precede apoptosis of human prostate epithelial cells. Urology. 2001;57(5):860-5.

[33] Kitaura H, Shinshi M, Uchikoshi Y, Ono T, Tsurimoto T, Yoshikawa H, et al. Reciprocal regulation via protein-protein interaction between c-Myc and p21 cip1/waf1/sdi1 in DNA replication and transcription. Journal of Biological Chemistry. 2000;275(14):10477-83.

[34] Lee YR, Chen M, Lee JD, Zhang J, Lin SY, Fu TM, et al. Reactivation of PTEN tumor suppressor for cancer treatment through inhibition of a MYC-WWP1 inhibitory pathway. Science. 2019;364(6441):eaau0159.

[35] Zhao Y, Cheng D, Wang S, Zhu J. Dual roles of c-Myc in the regulation of hTERT gene. Nucleic acids research. 2014;42(16):10385-98.

[36] Sasaki T, Kuniyasu H, Luo Y, Kitayoshi M, Tanabe E, Kato D, et al. AKT activation and telomerase reverse transcriptase expression are concurrently associated with prognosis of gastric cancer. Pathobiology. 2014;81(1):36-41.

[37] Guo S, Zhang L, Zhang Y, Wu Z, He D, Li X, et al. Long non-coding RNA TUG1 enhances chemosensitivity in non-small cell lung cancer by impairing microRNA-221-dependent PTEN inhibition. Aging (Albany NY). 2019;11(18):7553.

[38] Mandigo AC, Yuan W, Xu K, Gallagher P, Pang A, Guan YF, et al. RB/E2F1 as a Master Regulator of Cancer Cell Metabolism in Advanced DiseaseRB/E2F1 Regulates Cell Metabolism in Advanced Disease. Cancer discovery. 2021;11(9):2334-53.

[39] Matsumura I, Tanaka H, Kanakura Y. E2F1 and c-Myc in cell growth and death. Cell cycle. 2003;2(4):332-5.

[40] Brenner AK, Reikvam H, Lavecchia A, Bruserud Ø. Therapeutic targeting the cell division cycle 25 (CDC25) phosphatases in human acute myeloid leukemia—the possibility to target several kinases through inhibition of the various CDC25 isoforms. Molecules. 2014;19(11):18414-47.

[41] Berkovich E, Ginsberg D. ATM is a target for positive regulation by E2F-1. Oncogene. 2003;22(2):161-7.

[42] Wang C, Chen L, Hou X, Li Z, Kabra N, Ma Y, et al. Interactions between E2F1 and SirT1 regulate apoptotic response to DNA damage. Nature cell biology. 2006;8(9):1025-31.

[43] Malaney P, Palumbo E, Semidey-Hurtado J, Hardee J, Stanford K, Kathiriya JJ, et al. PTEN physically interacts with and regulates E2F1-mediated transcription in lung cancer. Cell Cycle. 2018;17(8):947-62.

[44] Jayasooriya RGPT, Dilshara MG, Molagoda IMN, Park C, Park SR, Lee S, et al. Camptothecin induces G2/M phase arrest through the ATM-Chk2-Cdc25C axis as a result of autophagy-induced cytoprotection: Implications of reactive oxygen species. Oncotarget. 2018;9(31):21744.

[45] Shen Y, Sherman JW, Chen X, Wang R. Phosphorylation of CDC25C by AMP-activated protein kinase mediates a metabolic checkpoint during cell-cycle G2/M-phase transition. Journal of Biological Chemistry. 2018;293(14):5185-99.

[46] Zhang X, Jia S, Yang S, Yang Y, Yang T, Yang Y. Arsenic trioxide induces G2/M arrest in hepatocellular carcinoma cells by increasing the tumor suppressor PTEN expression. Journal of cellular biochemistry. 2012;113(11):3528-35.

[47] Perdiguero E, Nebreda AR. Regulation of Cdc25C activity during the meiotic G2/M transition. Cell cycle. 2004;3(6):731-5.

[48] Masuda H, Fong CS, Ohtsuki C, Haraguchi T, Hiraoka Y. Spatiotemporal regulations of Wee1 at the G2/M transition. Molecular biology of the cell. 2011;22(5):555-69.

[49] Kim S, Lee H, Baek J, Cho Y, Kang H, Jeong J, et al. Activation of nuclear PTEN by inhibition of Notch signaling induces G2/M cell cycle arrest in gastric cancer. Oncogene. 2016;35(2):251-60.

[50] Zhang E, Yin D, Sun M, Kong R, Liu X, You L, et al. P53-regulated long non-coding RNA TUG1 affects cell proliferation in human non-small cell lung cancer, partly through epigenetically regulating HOXB7 expression. Cell death & disease. 2014;5(5):e1243-3.

[51] Bueno MJ, Gomez de Cedron M, Laresgoiti U, Fernández-Piqueras J, Zubiaga AM, Malumbres M. Multiple E2F-induced microRNAs prevent replicative stress in response to mitogenic signaling. Molecular and cellular biology. 2010;30(12):2983-95.

[52] Chen JH, Zhang P, Chen WD, Li DD, Wu XQ, Deng R, et al. ATM-mediated PTEN phosphorylation promotes PTEN nuclear translocation and autophagy in response to DNA-damaging agents in cancer cells. Autophagy. 2015;11(2):239-52.

[53] Stambolic V, MacPherson D, Sas D, Lin Y, Snow B, Jang Y, et al. Regulation of PTEN transcription by p53. Molecular cell. 2001;8(2):317-25.

[54] Yang G, Murashige DS, Humphrey SJ, James DE. A positive feedback loop between Akt and mTORC2 via SIN1 phosphorylation. Cell reports. 2015;12(6):937-43.

[55] Dan HC, Ebbs A, Pasparakis M, Van Dyke T, Basseres DS, Baldwin AS. Akt-dependent activation of mTORC1 complex involves phosphorylation of mTOR (mammalian target of rapamycin) by IκB kinase α (IKKα). Journal of Biological Chemistry. 2014;289(36):25227-40.

[56] Jung CH, Seo M, Otto NM, Kim DH. ULK1 inhibits the kinase activity of mTORC1 and cell proliferation. Autophagy. 2011;7(10):1212-21.

[57] Holczer M, Hajdú B, Lőrincz T, Szarka A, Bánhegyi G, Kapuy O. Fine-tuning of AMPK–ULK1–mTORC1 regulatory triangle is crucial for autophagy oscillation. Scientific reports. 2020;10(1):1-12.

[58] Wang J, Li J, Cao N, Li Z, Han J, Li L. Resveratrol, an activator of SIRT1, induces protective autophagy in non-small-cell lung cancer via inhibiting Akt/mTOR and activating p38-MAPK. OncoTargets and therapy. 2018;11:7777.

[59] Bhattacharya K, Maiti S, Mandal C. PTEN negatively regulates mTORC2 formation and signaling in grade IV glioma via Rictor hyperphosphorylation at Thr1135 and direct the mode of action of an mTORC1/2 inhibitor. Oncogenesis. 2016;5(5):e227-7.

[60] Kawata T, Tada K, Kobayashi M, Sakamoto T, Takiuchi Y, Iwai F, et al. Dual inhibition of the mTORC 1 and mTORC 2 signaling pathways is a promising therapeutic target for adult T-cell leukemia. Cancer science. 2018;109(1):103-11.

[61] Ghosh HS, McBurney M, Robbins PD. SIRT1 negatively regulates the mammalian target of rapamycin. PloS one. 2010;5(2):e9199.

[62] Sundin T, Peffley DM, Hentosh P. Disruption of an hTERT–mTOR–RAPTOR protein complex by a phytochemical perillyl alcohol and rapamycin. Molecular and cellular biochemistry. 2013;375(1):97-104.

[63] Alers S, Löffler AS, Wesselborg S, Stork B. Role of AMPK-mTOR-Ulk1/2 in the regulation of autophagy: cross talk, shortcuts, and feedbacks. Molecular and cellular biology. 2012;32(1):2-11.

[64] Liu Z, Sin KWT, Ding H, Doan HA, Gao S, Miao H, et al. p38β MAPK mediates ULK1-dependent induction of autophagy in skeletal muscle of tumor-bearing mice. Cell stress. 2018;2(11):311.

[65] Callus BA, Moujallad DM, Silke J, Gerl R, Jabbour AM, Ekert PG, et al. Triggering of apoptosis by Puma is determined by the threshold set by prosurvival Bcl-2 family proteins. Journal of molecular biology. 2008;384(2):313-23.

[66] Hershko T, Ginsberg D. Up-regulation of Bcl-2 homology 3 (BH3)-only proteins by E2F1 mediates apoptosis. Journal of Biological Chemistry. 2004;279(10):8627-34.

[67] Hemann M, Lowe S. The p53-BCL-2 connection. Cell death and differentiation. 2006;13(8):1256.

[68] Nakano K, Vousden KH. PUMA, a novel proapoptotic gene, is induced by p53. Molecular cell. 2001;7(3):683-94.

[69] Zhang CZ, Zhang JX, Zhang AL, Shi ZD, Han L, Jia ZF, et al. MiR-221 and miR-222 target PUMA to induce cell survival in glioblastoma. Molecular cancer. 2010;9(1):1-9.

[70] Murphy K, Ranganathan V, Farnsworth M, Kavallaris M, Lock RB. Bcl-2 inhibits Bax translocation from cytosol to mitochondria during drug-induced apoptosis of human tumor cells. Cell Death & Differentiation. 2000;7(1):102-11.

[71] Chipuk JE, Kuwana T, Bouchier-Hayes L, Droin NM, Newmeyer DD, Schuler M, et al. Direct activation of Bax by p53 mediates mitochondrial membrane permeabilization and apoptosis. Science. 2004;303(5660):1010-4.

[72] Tinahones FJ, Coín Aragüez L, Murri M, Oliva Olivera W, Mayas Torres MD, Barbarroja N, et al. Caspase induction and BCL2 inhibition in human adipose tissue: a potential relationship with insulin signaling alteration. Diabetes care. 2013;36(3):513-21.

[73] Gervais JL, Seth P, Zhang H. Cleavage of CDK inhibitor p21Cip1/Waf1 by caspases is an early event during DNA damage-induced apoptosis. Journal of Biological Chemistry. 1998;273(30):19207-12.

[74] Pawlowski J, Kraft AS. Bax-induced apoptotic cell death. Proceedings of the National Academy of Sciences. 2000;97(2):529-31.
